# Cytosine methylation contributes to the fitness of *Caulobacter* cells naturally expressing a Vsr-like protein

**DOI:** 10.1016/j.isci.2026.114749

**Published:** 2026-01-20

**Authors:** Noémie Matthey, Giorgia Wennubst, Nicolas Pellaton, Karolina Bojkowska, Julien Marquis, Justine Collier

**Affiliations:** 1 Department of Fundamental Microbiology, Faculty of Biology and Medicine, University of Lausanne, 1015 Lausanne, Switzerland; 2 Lausanne Genomic Technologies Facility, Faculty of Biology and Medicine, University of Lausanne, 1015 Lausanne, Switzerland

**Keywords:** Properties of biomolecules, Epigenetics, Microbial genomics, Transcriptomics

## Abstract

The role of methylated cytosines introduced into bacterial genomes by solitary DNA methyltransferases often remains elusive. Here, we show that the solitary cytosine methyltransferase ScmA of *Caulobacter crescentus* methylates thousands of YGCCGGCR motifs in its genome and that wild-type cells outcompete *ΔscmA* mutant cells. Transcriptomic and single-cell analyses reveal that a DNA damage response is turned on in a significant proportion of *ΔscmA* cells and that this response is strictly dependent on the Vsr-like protein VsrA. Although the *vsrA* gene is not genetically linked to *scmA* or to any other cytosine methyltransferase gene, VsrA is predicted to be a mismatch repair (MMR) endonuclease preventing 5mC-to-T mutations. In *ΔscmA* cells, VsrA may accidentally cause double-strand breaks (DSBs) during MMR, leading to reduced fitness. These findings suggest that *vsr*-like genes can stabilize solitary cytosine methyltransferase genes in bacterial genomes, potentially explaining their prevalence despite the frequent absence of detectable regulatory roles.

## Highlights

• Unmethylated DNA motifs can expose *Caulobacter* to genotoxic stresses and fitness losses
• Vsr-like proteins can be induced during bacterial DNA damage responses
• Vsr-like proteins can stabilize cytosine methyltransferase genes in bacterial genomes
• Cytosine methyltransferases may not be genetically linked with their cognate Vsr

## Graphical abstract

**Figure:**
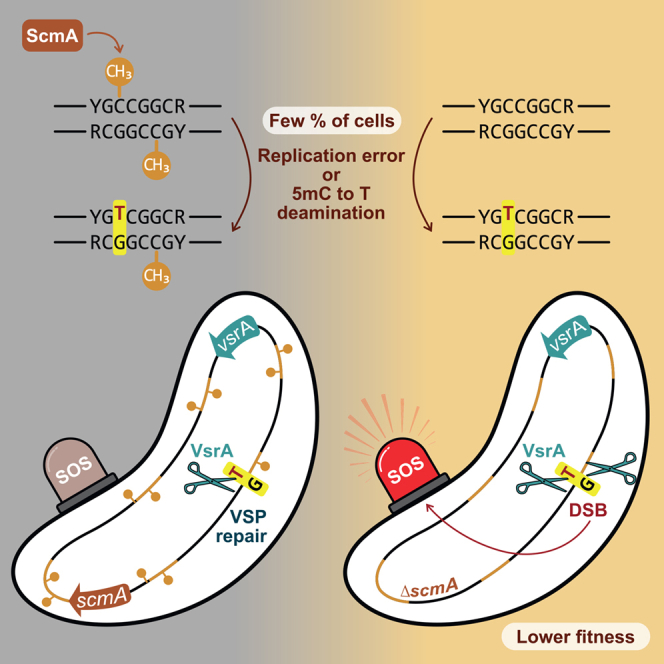

## Introduction

DNA methylation is a process that is widespread throughout all kingdoms of life. Such targeted chemical modifications of bases in genomes can modulate the binding of specific DNA binding proteins and thereby act as epigenetic signals that can influence gene expression.^1^^,^^2^^,^^3^ In eukaryotic genomes, C5-methyl-cytosines (5mC) were shown to be linked with a variety of human diseases including immunodeficiency and cancer.^4^ In bacteria, N6-methyl-adenines (6mA), 5mC, and/or N4-methyl-cytosines (4mC) are found in ∼93% of sequenced genomes and such methylated bases can represent up to 4% of all the bases of a bacterial genome.^5^^,^^6^ As in eukaryotes, methylated bases can influence gene expression through epigenetic processes in bacteria, but they can also play an important role in recognizing self-DNA during horizontal gene transfer (HGT) events and in protecting bacteria against phage infections.^1^^,^^7^

DNA methyltransferases (MTases) are the enzymes that can add methyl groups onto bases located in specific DNA motifs during a post-replicative process.^8^ In bacteria, most of the adenine/cytosine MTases are parts of so-called restriction-modification systems (RMS).^5^^,^^9^ In this case, the MTase gene is either located right next to a cognate restriction endonuclease (RE) gene or both activities are encoded by the same gene. The cognate RE recognizes the same DNA motif as the one methylated by the MTase but only when it is not methylated.^10^ Non-methylated motifs are often found on incoming DNA during HGT events or on phage genomes, which is how RMS can act as efficient phage defense mechanisms and limit HGT via RE-mediated DNA cleavage. Still, a significant proportion of the bacterial DNA MTases do not appear to be parts of RMS as they are not encoded by a gene located next to a predicted RE gene; in this case, they are called “solitary” (or “orphan”) DNA MTases.^5^^,^^11^

Bacterial solitary DNA MTases may have evolved from degenerate RMS or may have originated from incomplete transfers of mobile genetic elements (MGEs) as many RMS are found in such elements.^7^^,^^11^^,^^12^^,^^13^ Interestingly, even though they can then no longer act as RMS, a subset of these have acquired new roles in a diversity of biological processes such as DNA mismatch repair (MMR) and/or the regulation of chromosomal replication or gene expression.^6^^,^^7^^,^^14^^,^^15^^,^^16^^,^^17^ Bacterial epigenetic processes can, for example, regulate cell cycle progression, developmental pathways, or virulence. It is, however, interesting to note that most of the solitary DNA MTases that have been shown to play a regulatory role in bacteria are adenine MTases generating 6mA epigenetic signals.

Despite their frequent occurrence, the exact biological role of solitary cytosine MTases creating 5mC bases in bacterial genomes usually remains elusive^7^ except for rare examples from *Gammaproteobacteria* and from Group B *Streptococci* (GBS). First, the Dcm cytosine MTase of *Escherichia coli* (*E.coli*) has been shown to influence stationary phase gene expression, thereby improving the fitness of cells under these conditions.^18^^,^^19^^,^^20^ Second, the VchM cytosine MTase of *Vibrio cholerae* (*V. cholerae*) has been shown to repress the expression of chaperonin-encoding genes (*groESL-2*), playing a role in the tolerance of this bacterium to aminoglycosides^21^ and maybe also in its capacity to infect its hosts.^22^ An example is the Dcm cytosine MTase of GBS that has a major impact on its transcriptome, especially concerning genes encoding proteins involved in carbohydrate transport and metabolism.^23^

The frequent occurrence of 5mC bases in bacterial genomes is even more intriguing if we consider that these have been shown to be significantly more mutagenic than non-methylated bases, potentially constituting a threat for genome integrity.^24^^,^^25^ Indeed, 5mC bases that get accidentally deaminated into T can generate C-to-T mutations if resulting TG mismatches are not detected and repaired on time before the next round of DNA replication. In addition, the spontaneous deamination of 5mC occurs at a higher rate than non-modified C, generating C-to-T transition hot-spots at DNA motifs methylated by cytosine MTases in genomes.^26^ Thus, it has been proposed that cytosine MTases may have a significant impact on the rate of evolution of bacterial genomes.^27^^,^^28^ Still, some bacteria have developed dedicated repair systems, known as very short patch (VSP) repair systems, that can detect and then repair TG mismatches in DNA motifs that were initially methylated by cytosine MTase(s).^24^^,^^29^^,^^30^ In these cases, a so-called Vsr endonuclease detects the TG mismatch in the target motif and then catalyzes a nick of the DNA strand containing the T to initiate its removal by a helicase and its replacement with a C through the resynthesis of a short DNA patch by the DNA Pol I. This bacterial VSP repair process was first discovered in *E. coli* where Vsr^Ec^ detects TG mismatches in CCWGG motifs initially methylated by Dcm.^24^^,^^30^ In this case, the *vsr* gene is located downstream of its cognate *dcm* gene and co-transcribed from the same operon.^31^ Later, several other Vsr proteins from a variety of *Neisseria gonorrhoeae*^32^^,^^33^ and *Neisseria meningitidis* strains^34^ have been characterized. These bacteria often have more than one cytosine MTase and Vsr proteins, but *vsr* genes still appear to stay genetically linked with (sometimes truncated) cytosine MTase genes (solitary or parts of RMS). *In vitro* experiments showed, however, that a few of these Vsr proteins can recognize TG mismatches in relatively degenerate motifs,^32^ suggesting that they may be functionally linked with more than one cytosine MTase even if not genetically linked. In some cases, strains carry cytosine MTase genes but no *vsr*-like genes anywhere on the genome.^9^

Despite recent progress in the field of bacterial epigenetics/epigenomics, essentially nothing is known about the biological role and the impact of solitary cytosine MTases in the very diverse class of *Alphaproteobacteria* despite their high prevalence.^5^^,^^9^ To address this question, the *Caulobacter crescentus Alphaproteobacterium* model appears a prime choice, especially since an analysis of its methylome at different times of its cell cycle has shown that two different DNA motifs carry 5mC bases^35^ and since we have already shown that 6mA bases on this genome can play a critical role in cell cycle regulation and survival.^15^^,^^36^^,^^37^ Consistent with the existence of two DNA motifs carrying 5mC bases, *C. crescentus* encodes two predicted cytosine MTases: CCNA_03741 and CCNA_01085 (also known as M.CcrNAIV and M.CcrNAV, respectively, in REBASE^38^). Their genes are not genetically linked with a gene annotated to encode an obvious RE and can thus both be classified as solitary DNA MTases.^35^ Here, we dissected the potential influence of the solitary cytosine MTase CCNA_01085, now named ScmA, in *C. crescentus*. We constructed a *ΔscmA* mutant and used it to study the impact of ScmA-dependent methylation on *C. crescentus* phenotypes, on its fitness and on its transcriptome. These analyses revealed that ScmA protects cells from DNA damage that can be induced by the CCNA_02930 Vsr-like protein that is now named VsrA. For this reason, wild-type cells largely outcompete *ΔscmA* cells during standard growth conditions. Interestingly, the *vsrA* gene is not genetically linked with the *scmA* gene despite the functional link that we discovered, revealing an original gene positioning architecture compared to previously characterized systems.

## Results

### ScmA methylates the first cytosine in YGCCGGCR motifs

**Figure 1:**
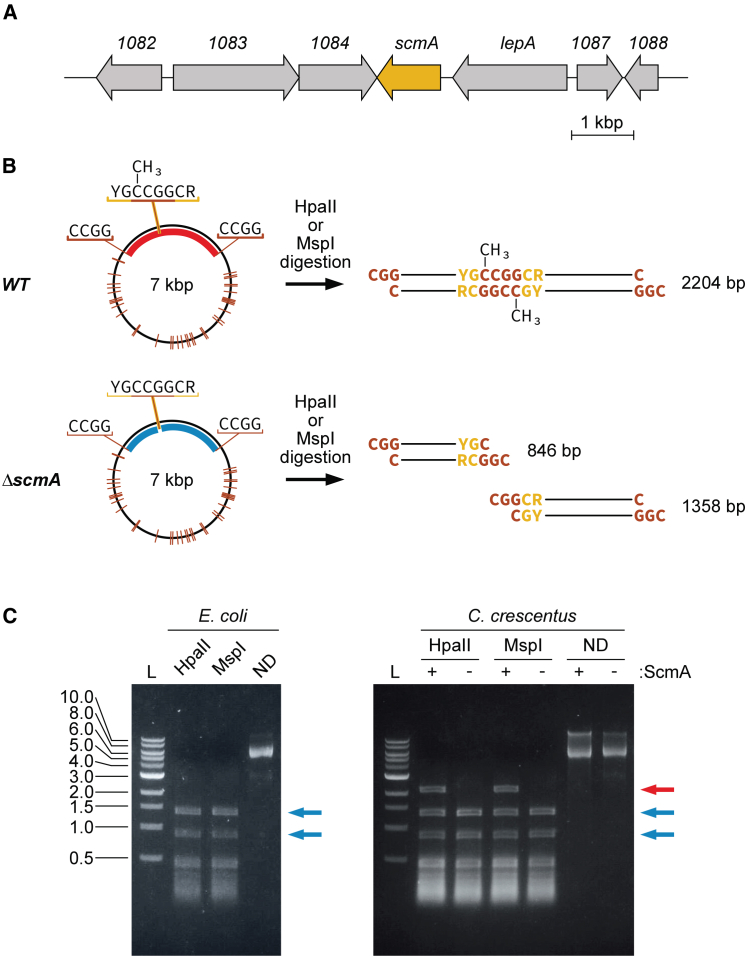
ScmA methylates the first cytosine in YGCCGGCR motifs (A) Schematic showing the organization of genes around *scmA* (*CCNA_01085*) on the *C. crescentus* NA1000 chromosome. Genes of unknown function are shown using their *CCNA* numbers. (B) Map of the pBX-1motif plasmid (left) showing the position of the unique YGCCGGCR motif and of the 40 other CCGG motifs (orange lines). These motifs are all cut by the 5mC-sensitive HpaII and MspI restriction endonucleases. The right images show the size (bp) of the expected large restriction fragments following digestion with HpaII or MspI in *WT* or *ΔscmA C. crescentus* cells if ScmA can methylate the first cytosine of the unique YGCCGGCR motif found on that plasmid. (C) Image of an agarose gel showing the size of the restriction fragments detected after a digestion of the pBX-1motif plasmid using HpaII, MspI or no enzyme (non-digested; ND). Prior to digestion, pBX-1motif was extracted from *E. coli* TOP10 cells (left) or from *WT* (JC450; +) or *ΔscmA* (JC2005; -) *C. crescentus* cells (right). Blue arrows highlight the two large restriction fragments (846 and 1,358 bp) obtained if the first C in the YGCCGGCR motif is not methylated (HpaII and MspI can then cut the CCGG motif included in that larger motif); the red arrow highlights the unique large restriction fragment obtained (2,204 bp) if the first C in the YGCCGGCR motif is methylated (HpaII and MspI can then not cut the CCGG motif included in that larger motif). L: DNA ladder (kbp).

ScmA (CCNA_01085) was predicted to be a solitary type II cytosine MTase since its gene is neither genetically linked with a predicted RE-encoding gene (Figure 1A), nor encoding a putative endonuclease activity (like RMS IIG).^9^^,^^35^^,^^39^ Consistent with this, we easily constructed a *ΔscmA* mutant strain, demonstrating that ScmA is not essential to protect the *C. crescentus* genome against a lethal RE activity as expected for MTases that are parts of RMS.

Our previous *C. crescentus* methylome analysis suggested that ScmA may methylate the first C in 5′-YGCCGGCR-3′ motifs since this was the only remaining detected DNA motif containing 5mC that is not methylated by the second cytosine MTase (CCNA_03741) found in this bacterium.^35^ To confirm this by-default prediction, we used an experimental method based on using 5mC-dependent RE to digest DNA with a motif that is expected to contain a modified 5mC (Figure 1B).^28^ We first constructed a plasmid (named pBX-1motif) containing a single YGCCGGCR motif and introduced it into *E. coli* cells (control) or into *WT* and *ΔscmA C. crescentus* cells. The pBX-1motif plasmid was then extracted from each strain and its capacity to be restricted by HpaII or MspI was tested *in vitro*. HpaII cuts CCGG motifs but only when none of its cytosines are methylated, while MspI cuts non-methylated CCGG motifs (like HpaII) or CCGG motifs with 5mC at the second position.^38^ When the pBX-1motif plasmid was extracted from *E. coli* cells that do not encode a cytosine MTase that can methylate a CCGG motif, the digestion pattern consisted of two large restriction fragments of ∼846 and ∼1,358 bp (Figure 1C) together with many smaller ones, exactly as expected if neither of the two C found in the unique YGCCGGCR motif engineered on this plasmid are methylated (Figure 1B). Instead, when the pBX-1motif plasmid was extracted from *WT C. crescentus* cells, we observed a third large restriction fragment of ∼2,204 bp = (846 + 1,358 bp) when it was digested with either HpaII or MspI (Figure 1C), demonstrating that the first cytosine of the YGCCGGCR located inside this larger restriction fragment was often methylated (5mC), protecting it from digestion by HpaII and MspI (Figure 1B). Importantly, when this plasmid was instead extracted from *ΔscmA C. crescentus* cells, the protected ∼2,204 bp fragment could no longer be detected (Figure 1C), demonstrating that ScmA is indeed the cytosine MTase that can methylate the first cytosine of the unique YGCCGGCR motif of this test plasmid in *WT C. crescentus* cells.

It is noteworthy that we could still detect the ∼1,358 and ∼846 bp restriction fragments, although at lower levels, following the digestion of the test plasmid extracted from *WT C. crescentus* cells (Figure 1C). This observation suggests that a certain proportion (estimated at ∼1/3) of the plasmid molecules were not efficiently methylated by ScmA in the *WT C. crescentus* population from which the plasmid was extracted. This is reminiscent of a previous observation, when we could only detect 5mC in ∼76% of the sequencing reads containing YGCCGGCR motifs during Single Molecule Real time (SMRT)-sequencing analyses after a Tet conversion treatment of the *WT C. crescentus* genome commonly used to detect 5mC modifications.^35^ Thus, these two independent observations using different methods suggest that some *C. crescentus* cells may not contain enough ScmA to methylate all the YGCCGGCR motifs found in their genome or that ScmA may not be available to methylate these motifs in a subset of *WT* cells.

Looking at the *C. crescentus* genome sequence (NA1000 strain GenBank ID: CP001340.1), we found 3,054 YGCCGGCR motifs that could be methylated by ScmA, a slightly higher number than expected randomly for the *C. crescentus* genome of ∼4 Mbp and ∼67% of G/C content. Still, only 91 (2.98%) of these motifs were mapped in intergenic (IG) regions, demonstrating a significant under-representation since IG regions cover ∼9.6% of the genome.

### Wild-type cells out-compete *ΔscmA cells*

**Figure 2:**
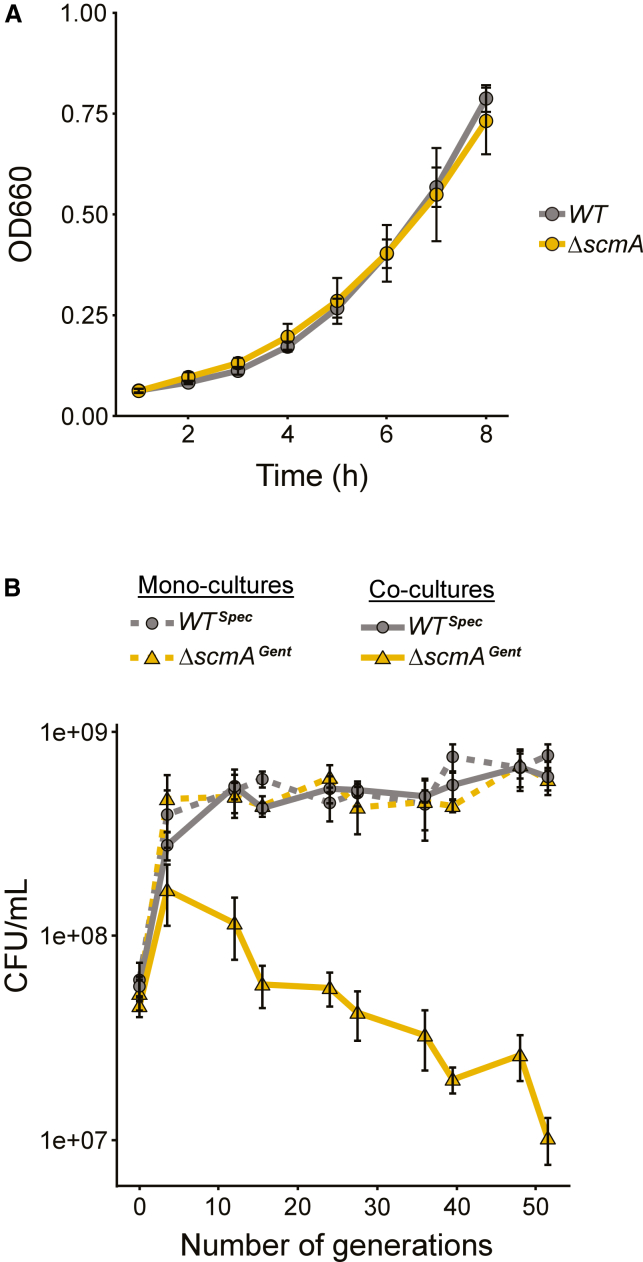
Wild-type *C. crescentus* cells outcompete isogenic *ΔscmA* cells during competition experiments (A) Growth curves of *WT* (JC450) and *ΔscmA* (JC2005) cells cultivated in complex PYE medium. Cells were first cultivated overnight in PYE medium (reaching stationary phase) and cultures were then diluted back into fresh PYE medium to reach an OD_660nm_–0.05 at time 0. The OD_660_ was then measured every hour for 8 h. The values plotted in these growth curves correspond to the average measurements from at least three independent biological experiments. (B) Monocultures (dashed lines) and co-cultures (solid lines) of *WT*^*Spec*^ (JC2985) and/or Δ*scmA*^*Gent*^ (JC2984) cells were cultivated in complex PYE medium for more than 50 generations (the generation time of these strains is close to 2 h under these conditions). Before each regular dilution, spectinomycin-resistant (*WT*^*Spec*^) and gentamycin-resistant (Δ*scmA*^*Gent*^) colony forming units (CFUs) per mL of (co-)cultures were measured on antibiotic-containing PYEA plates. The plotted values correspond to average measurements from four independent biological replicates. In (A) and (B), error bars correspond to standard deviations (±SD).

To characterize the impact of DNA methylation by ScmA in *C. crescentus*, we first compared the growth and the morphology of *WT* and *ΔscmA* cells. The growth rates of the two strains cultivated in exponential were very similar in PYE complex medium (Figure 2A, 104 minutes compared to 121 min for *WT* and *ΔscmA* cells, respectively) and in M2G minimal medium (Figure S1, 145 minutes compared to 150 min minutes for *WT* and *ΔscmA* cells, respectively). Microscopy analyses of exponentially growing cells cultivated in M2G medium however revealed that *ΔscmA* cells were more often unusually elongated (1.76-fold more frequently with a cell length ≥ 3.5 μm) than *WT* cells (Figure S2). The cell cycle of these cells may be arrested, or they may be subject to a stress that blocks their division. These elongated cells represented an only limited proportion (6.09%) of the cells in the *ΔscmA* population, suggesting a rather heterogeneous or transient response to the lack of ScmA.

Considering these very subtle phenotypes, we next decided to compare the fitness of *WT* and *ΔscmA* cells when growing and competing in PYE medium over a prolonged period (∼50 generations). To differentiate the strains from one another, we added spectinomycin or gentamycin resistance cassettes as selective markers in the genomes of *WT* and Δ*scmA* cells, obtaining the so-called *WT*^*Spec*^, *WT*^*Gent*^, and *ΔscmA*^*Gent*^ strains. To evaluate the relative maintenance of each strain in mixed populations over time, we measured spectinomycin- and gentamycin-resistant colony forming units (CFUs)/mL of cultures over time for ∼40–-50 generations making regular dilutions of these co-cultures (Figures 2B and S3 as controls). Mono-cultures of each strain were also tracked following the same experimental set-up. Strikingly, when *WT*^*Spec*^ and *ΔscmA*^*Gent*^ strains were cultivated together, *WT*^*Spec*^ cells rapidly outcompeted *ΔscmA*^*Gent*^ cells (Figure 2B), which was not the case when *WT*^*Spec*^ cells were cultivated with control *WT*^*Gent*^ cells (Figure S3), demonstrating a strong loss of fitness associated with the loss of ScmA in *C. crescentus*. Indeed, the *ΔscmA*^*Gent*^ cells in the mixed population were rapidly replaced by the *WT*^*Spec*^ cells, with ∼10-fold more *WT*^*Spec*^ cells than *ΔscmA*^*Gent*^ cells after ∼24 generations and ∼60-fold more after ∼50 generations. Thus, *scmA* is not just a cryptic gene that could be easily lost by *C. crescentus* in natural settings when spontaneous mutants/variants usually first compete with their *WT* ancestors before they may find they own favorite niche to propagate.

### Impact of ScmA on the *C. crescentus* transcriptome

**Figure 3:**
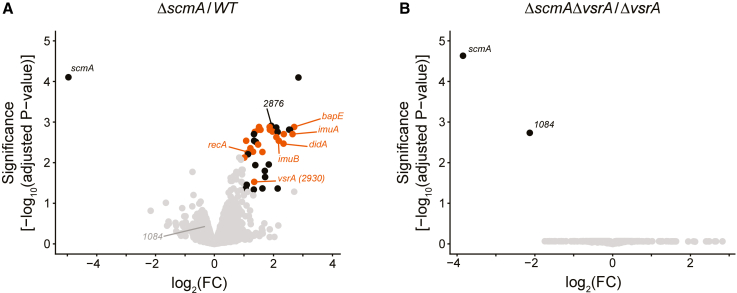
Genes known to be activated in response to DNA damage in *C. crescentus* are activated in Δ*scmA* cells and in a VsrA-dependent manner Volcano plots showing RNA-seq results comparing the transcriptomes of *C. crescentus* cells carrying or not the *scmA* gene and cultivated exponentially in M2G medium. FC indicates the fold-change when comparing mRNA levels in cells that do not carry *scmA* compared with cells that do carry *scmA*. Each dot corresponds to one gene. Gray dots correspond to genes that are not considered as differentially regulated (adjusted *p* value > 0.05 and/or −1<log_2_FC < 1). Black and red dots correspond to the 41 genes that are differentially regulated (adjusted *p* value < 0.05 and log_2_FC > 1 or log_2_FC < −1). Red dots correspond to the 21 differentially regulated genes that are known to be induced during *C. crescentus* DNA damage responses.^40^^,^^41^^,^^42^ Several non-annotated genes are shown with their *CCNA* numbers. Glimma volcano plots (interactive HTML graphics) are also available as supplemental information. Adjusted *p* values were calculated based on three independent biological replicates for each strain. (A) Volcano plot comparing the transcriptomes of *WT* (JC450) and *ΔscmA* (JC2005) cells. (B) Volcano plot comparing the transcriptomes of *ΔvsrA* (JC2540) and *ΔscmA ΔvsrA* (JC2542) cells.

Considering that ScmA is a solitary DNA MTase (Figure 1) and that 5mC bases can potentially affect gene expression, we first hypothesized that methylation by ScmA may have an impact on the expression of *C. crescentus* genes controlled by promoters that can get methylated by ScmA. As mentioned above, 91 of the YGCCGGCR motifs found on the *C. crescentus* genome are found in IG regions that often contain promoter regions. Thus, to shed light on why *WT* cells can outcompete *ΔscmA* cells (Figure 2B), we decided to compare the transcriptomes of *WT* and *ΔscmA* cells cultivated in exponential phase in M2G medium. RNA sequencing (RNA-seq) experiments revealed that the mRNA levels of 41 genes were significantly different between the two strains (adjusted *p* value < 0.05 and log_2_FC ≥ 1 or ≤ −1 in Figure 3A and Table S4). Among these, the only gene that was significantly downregulated in *ΔscmA* cells compared to *WT* cells was *scmA*. Strikingly, when looking at the remaining 40 genes, which were all upregulated in Δ*scmA* cells compared to *WT* cells, we found an important over-representation (8.4-fold) of the Cluster of Orthologous Group (COG) category L corresponding to “replication, recombination, and repair” (Figure S4). A more detailed analysis (Figure 3A and Table S4) revealed that 52% of these upregulated genes (21 genes) had been previously shown to be induced in response to DNA damage in *C. crescentus*.^40^^,^^41^^,^^42^ Examples include the well-conserved *recA* gene (2.2-fold induction in *ΔscmA* compared to *WT* cells), the genes encoding the alternative DNA polymerases *imuA* (6.3-fold induction) and *imuB* (4.6-fold induction) and the gene encoding the apoptosis endonuclease *bapE* (6.6-fold induction). Most of these genes have a LexA-binding box in their promoter region and belong to the SOS-dependent DNA damage response pathway.^40^^,^^41^^,^^42^^,^^43^^,^^44^ Still, a few others, including the gene encoding the cell division inhibitor *didA* (5.1-fold induction), belong to other known SOS-independent DNA damage response pathways that are independent of RecA/LexA in *C. crescentus*.^40^ Thus, this transcriptome analysis indicated that DNA damage may take place in at least a subset of *ΔscmA* cells, inducing a general DNA damage response.

We also searched for genes that were significantly upregulated in *ΔsmcA* cells and that contained at least one YGCCGGCR motif (ScmA target) in their promoter region (250 bp upstream of each open reading frame). Eight genes were found (8/40) (Table S4), including three that also belonged to the LexA regulon.^40^ Thus, altogether, the results of the transcriptome analyses suggested that maximum 5–8 genes may be regulated through the addition of 5mC epigenetic marks onto their promoter region by ScmA, or, alternatively, that all the observed misregulated genes may simply be linked with the DNA damage response that seemed to be turned on in *ΔscmA* cells.

### An SOS response is turned on in a subset of *ΔscmA* cells and in a MutL-independent manner

Since we identified many genes known to be activated during the *C. crescentus* SOS response that were also upregulated in *ΔscmA* cells, we aimed at verifying that an SOS response is indeed turned on in such mutant cells and then also at testing whether this response is homogeneous or heterogeneous in the *ΔscmA* population.

**Figure 4:**
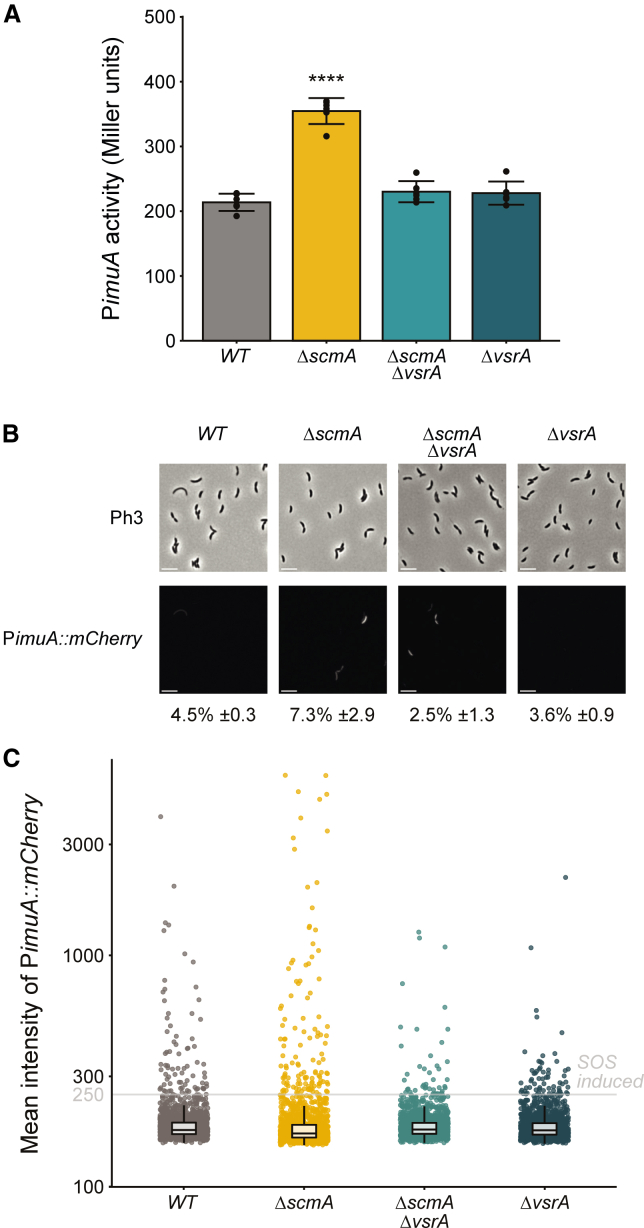
A VsrA-dependent SOS response is turned on in a subset of *ΔscmA* cells (A) β-galactosidase assays showing the VsrA-dependent activation of an SOS response in *ΔscmA* populations of cells. The pP*imuA*:*lacZ*290 plasmid (SOS reporter) was introduced into *WT* (JC450), *ΔscmA* (JC2005), *ΔvsrA* (JC2540) or *ΔscmA ΔvsrA* (JC2542) *C. crescentus* cells. The resulting strains were cultivated in PYE complex medium until cultures reached 0.25 < OD_660nm_ < 0.3 and β-galactosidase assays were then performed. The plotted promoter activity values (in Miller units) are averages of six independent biological replicates, each with two technical replicates. Error bars correspond to standard deviations (±SD). Statistically significant differences compared to *WT* cells using a Student’s *t* test is indicated as follows: ∗*p* < 0.05; ∗∗∗∗*p* <0.0001). (B) Single-cell fluorescence microscopy assays showing that a VsrA-dependent SOS response is turned on in a subset of *ΔscmA* cells in clonal populations. The P*imuA::mCherry* reporter was integrated at the *imuA* locus on the genome of *WT* (giving JC3104), *ΔscmA* (giving JC3105), *ΔvsrA* (giving JC3107), or Δ*scmA ΔvsrA* (giving JC3109) cells. The resulting strains were cultivated in exponential phase in PYE complex medium. Phase contrast (Ph3) and mCherry images of representative cells are shown. Scale bars, 2 μm. (C) Quantification of the cytoplasmic fluorescence intensity of cells in populations from (B). The average cytoplasmic fluorescence intensity (arbitrary units) of minimum 1,600 cells of each strain/culture (4 biological replicates of each with minimum 400 cells/replicate) is shown: the boxes indicate the interquartile range with the center representing the median. Dots above the “SOS” threshold represented by the gray line were used to estimate the percentage of cells in each population displaying an obvious SOS response, as indicated under each microscopy image in (B).

As a first whole-population reporter of this putative SOS response, we used a plasmid carrying a transcriptional fusion between the *imuA* promoter and the *lacZ* ORF (P*imuA::lacZ*). ImuA is an alternative *trans*-lesion DNA polymerase that is turned on in a LexA-dependent manner in response to DNA damage in *C. crescentus*.^43^ This plasmid was introduced into *WT* and *ΔscmA* cells and the average activity of the P*imuA* promoter in each cell population was measured by β-galactosidase assays using exponentially growing cells cultivated in PYE medium. The results showed that the LexA-dependent P*imuA* promoter was significantly more active in the *ΔscmA* than in the *WT* cell population (1.66-fold induction, Figure 4A), confirming that a higher than basal SOS response can be detected in *ΔscmA* cells. However, compared to *WT* cells that were exposed to severe external DNA damaging conditions, such as UV or mitomycin C,^43^ this activation remained relatively modest. This observation could be the result of either an SOS response that was turned on at low intensity in all the *ΔscmA* cells, or of a strong SOS response found only in a subset of the *ΔscmA* cells in the population.

To distinguish between these two hypotheses, we constructed a single-cell fluorescent reporter of the SOS response corresponding to a P*imuA::mCherry* construct integrated at the native *imuA* chromosomal locus of *WT* or *ΔscmA* cells. Using this reporter, fluorescence microscopy analyses were used to test whether the SOS response that is turned on in *ΔscmA* cells is homogeneous or heterogeneous in the clonal population. Strikingly, these single-cell assays showed that only a subset of the cells appeared obviously more fluorescent than others in the *ΔscmA* population (Figures 4B and 4C), indicating that the weak SOS response detected from whole cell populations (Figure 4A) mostly results from a strong SOS induction in a sub-population of cells, rather than from a weak induction in all the cells of the population. This result was further verified using a second single cell fluorescent reporter (P*bapE::mCherry*) of the SOS response (Figure S5) that gave very similar results.

Interestingly, this observation is reminiscent of what was previously found for *E. coli dam* mutants.^45^ Dam is also a solitary DNA MTase, but it methylates adenines in GATC motifs on the *E. coli* chromosome. In *E. coli*, 6mA bases play an important role during the DNA MMR process, when they are used by the MutH endonuclease to distinguish the newly synthesized DNA strand containing the mismatch that needs to be nicked by MutH during the MMR process.^46^ In *Δdam* cells, 6mA signals are missing, and this results in the generation of double-strand breaks (DSBs) by MutH that then nicks the two DNA strands instead of one when it tries to repair mismatches from replication errors. Consequently, an SOS response is regularly detected in *Δdam* cells.^47^^,^^48^ Since *C. crescentus* does not have Dam/MutH homologs and since ScmA is also a solitary DNA MTase, we hypothesized that cytosine methylation by ScmA may play a similar role in strand discrimination during the MMR process in *C. crescentus*, potentially explaining why an SOS response is accidentally turned on in a subset of *ΔscmA* cells. If that was the case, one would expect that the SOS response turned on in *ΔscmA* cells would be suppressed in MMR-defective cells. In *C. crescentus*, we recently showed that two proteins contribute to the MMR process: MutS that recognizes the mismatches and MutL that acts as the endonuclease that nicks the newly synthesized DNA strand containing the replication error to initiate the repair process^49^ (Figure S6). To test if MutL may be responsible for the SOS response turned on in *ΔscmA* cells, we compared the activity of the SOS reporter (P*imuA::lacZ*) in *ΔscmA* and *ΔscmA ΔmutL* mutant cells. β-galactosidase assays showed that the SOS response was essentially identical in both strains (Figure S7), showing that the MutL endonuclease is not responsible for the SOS response that is turned on in *ΔscmA* cells. Thus, DNA methylation by ScmA most likely does not play a role in the *C. crescentus* MMR process as does Dam in *E. coli*.

### VsrA is responsible for the SOS response turned on in a subset of *ΔscmA* cells

Considering that the MutL endonuclease is not responsible for the SOS response that is turned on in *ΔscmA* cells, we searched for other intracellular sources of potential DNA damage in these cells. Interestingly, our transcriptome analysis revealed that two genes annotated to encode Vsr-like endonucleases were significantly upregulated in *ΔscmA* cells compared to *WT* cells (Figure 3A and Table S4): *CCNA_02876* (3.8-fold induction) and *CCNA_02930* (2.6-fold induction). To test whether these putative endonucleases may play a role in the SOS response that was observed in *ΔscmA* cells, we deleted one or the other gene in the *WT* and *ΔscmA* strains and then introduced an SOS reporter (P*imuA::lacZ*) into these strains. Strikingly, subsequent β-galactosidase assays revealed that the SOS response was back to basal levels (as *WT*) in *ΔscmA* cells that lacked CCNA_02930 (Figure 4A), while the absence of CCNA_02876 had no impact (Figure S8). We also confirmed this result using the two fluorescent single-cell reporters P*imuA::mCherry* and P*bapE::mCherry* (Figures 4B, 4C, and S5). Altogether, these results demonstrate that the CCNA_02930 putative endonuclease is fully responsible for the SOS response that is turned on in a subset of *ΔscmA* cells.

**Figure 5:**
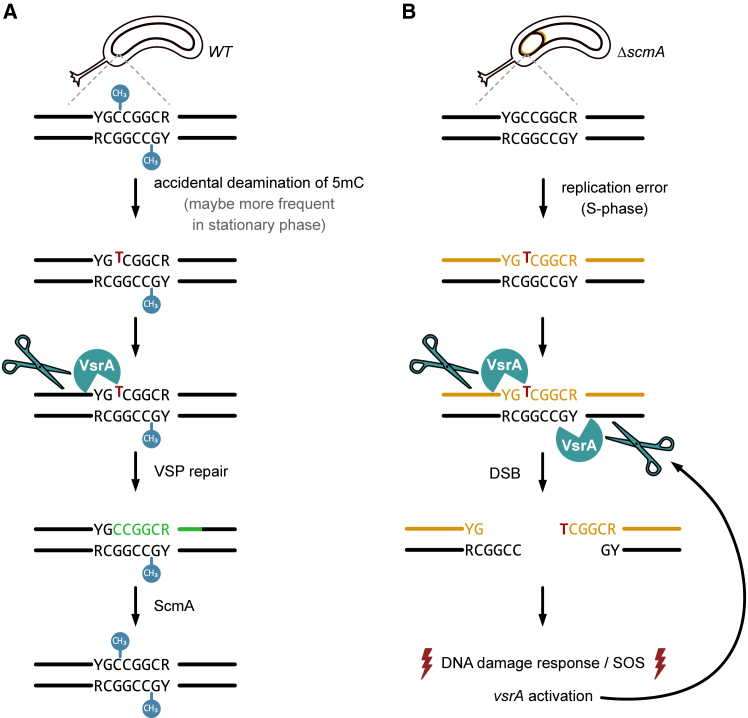
Model for a VsrA-dependent VSP repair process in *C. crescentus* (A) Schematic showing how TG mismatches arising in YGCCGGCR motifs methylated by ScmA (from the accidental 5mC deamination into T) may be repaired in VsrA-dependent manner in *WT C. crescentus* cells. The bases replaced during this VSP repair process are shown in green. This model is based on the Vsr-dependent VSP repair mechanism discovered in *E. coli* to repair TG mismatches in DNA motifs methylated by Dcm.^29^ (B) Schematic showing how VsrA may generate accidental double-strand breaks (DSB) if it tries to repair TG mismatches (from DNA replication errors) in non-methylated YGCCGGCR motifs in *ΔscmA C. crescentus* cells. This model is inspired from what was shown to happen when MMR proteins try to repair DNA mismatches in *E. coli Δdam* mutant cells.^47^^,^^48^ The newly synthesized DNA strand is shown in orange in this schematic.

Looking more carefully at the CCNA_02930 protein sequence (Figure S9), we found that it belongs to the PD-(D/E)XK superfamily of endonucleases^50^ such as the Vsr protein of *E. coli* and that it contains a DUF559 domain, which is often found in Vsr-like endonucleases in a diversity of bacteria. On top of that, a comparison of its predicted 3D structure with that of the well-known *E. coli* Vsr protein^51^ showed that a large part of CCNA_02930 displays similarities. We therefore decided to rename CCNA_02930 VsrA and hypothesized that it may be a VSP repair protein detecting TG mismatches (Figure 5A) like the *E. coli* Vsr protein.^52^

### Cytosine methylation by ScmA does not impact gene expression in the absence of VsrA and no more affects the fitness of *C. crescentus*

Finding that many SOS response genes were turned on in the *ΔscmA* mutant (Figure 3A) complicated our initial goal of determining whether ScmA-dependent methylation may regulate the expression of specific genes in *C. crescentus*. Luckily, observing that the SOS response appeared suppressed in the *ΔscmA ΔvsrA* double mutant (Figure 4) unlocked a novel strategy enabling us to potentially decouple the VsrA-induced SOS response from the potential direct impact that ScmA methylation may have on the regulation of gene expression. To address the initial question, we then simply performed RNA-seq experiments comparing the transcriptomes of single *ΔvsrA* and double *ΔscmA ΔvsrA* mutant cells (Figure 3B) in the same growth conditions as before (Figure 3A). Strikingly, our results showed that the only gene that was still significantly misregulated (adjusted *p* value < 0.05 and log_2_FC ≤ −1) in the *ΔscmA ΔvsrA* strain compared to the *ΔvsrA* strain, beyond the expected *scmA* gene, was the *CCNA_01084* gene (Figure 3B and Table S5), which is located right next to *scmA* on the *C. crescentus* chromosome (Figure 1A). Even though this gene is not in the same operon as *scmA* (they are in opposite directions), it is very likely that the deletion of *scmA* that we engineered simply interfered with the normal expression of the nearby *CCNA_01084*. Importantly, its promoter region (250 bp upstream of the ORF) does not include a YGCCGGCR motif (Table S5) ruling out a potential direct impact of ScmA-based methylation on its expression. Taken together, this transcriptome analysis demonstrated that all the genes that were induced in *ΔscmA* cells were linked with the presence of VsrA. Since VsrA is not predicted to be a transcription factor but rather a putative endonuclease (Figure S9), and since a majority of the genes that were initially induced are known to be activated in response to DNA damage (and without YGCCGGCR motifs in their promoter region) (Figure 3A and Table S4), we hypothesize that VsrA damages the *C. crescentus* chromosome in a subset of *ΔscmA* cells thereby turning on the observed heterogeneous DNA damage response (Figure 4). This important result also indicates that the methylation of cytosines by ScmA on the *C. crescentus* chromosome most likely does not play a role in the regulation of gene expression, at least in the chosen growth conditions.

Considering this information, we hypothesized that the loss of fitness of Δ*scmA* cells compared to *WT* cells (Figure 2B) may then be simply linked to the VsrA-dependent DNA damage response induced in a subset of the mutant cells. To test this, we compared the fitness of Δ*scmA ΔvsrA*^*Gent*^ double mutant cells with that of *WT*^*Spec*^ cells during competition experiments for over ∼50 generations (Figure S10). Remarkably, the absence of VsrA completely abolished the deleterious fitness impact linked with the absence of ScmA (Figure S10).

Altogether, it is thus very likely that the VsrA-dependent SOS response that was turned on in *ΔscmA* cells was responsible for the loss of competitiveness of these mutant cells compared to *WT* cells.

### VsrA forms frequent foci in stationary phase cells

As suggested earlier, the *C. crescentus* VsrA protein may be a VSP repair protein*.* Considering the functional link that we discovered between ScmA and VsrA and the analogy with the Dcm/Vsr pair in *E. coli*, we now hypothesize that VsrA may recognize TG mismatches arising from the spontaneous deamination of 5mC bases into T in the YGCCGGCR motif methylated by ScmA in *WT* cells, to initiate their repair before they become stable C-to-T mutations during the next round of DNA replication (Figure 5A). In this model, VsrA would only nick the non-methylated DNA strand of YGCCGGCR motifs containing a mis-matched T (instead of a 5mC) opposite a G to start the VSP repair process in *WT* cells.

What would then happen in a *ΔscmA* cell if a T is accidentally mis-incorporated instead of a C during DNA replication errors (by the replicative DNA Pol III or by the alternative error-prone DNA polymerases that are turned on during the SOS response) at the third position of a non-methylated YGCCGGCR motif? One can predict that VsrA could end up nicking both DNA strands as neither of these two DNA strands are methylated in such cells (Figure 5B), exactly as MutH does in *E. coli* Δ*dam* cells during the MMR process.^47^^,^^48^ If this was the case, DSBs would be generated in a subset of the cells in the population (every time a T is accidentally incorporated at the third position of one of the 3054 YGCCGGCR motifs on the *C. crescentus* chromosome) potentially inducing an SOS response to repair these DSBs, which is consistent with our findings (Figures 4B and 4C).

**Figure 6:**
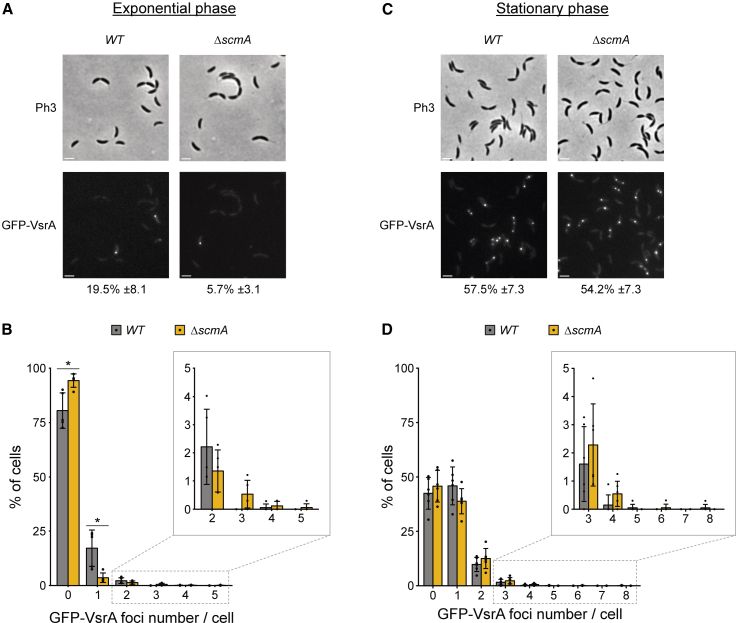
GFP-VsrA forms foci in a majority of stationary phase *C. crescentus* cells The native *vsrA* gene was replaced by a *gfp-vsrA* construct in *WT* and *ΔscmA* cells, giving the JC2860 and JC2861 strains, respectively. These strains were then cultivated in exponential (A and B) or stationary phase (C and D) in complex PYE medium before cells were imaged with a fluorescence microscope. Phase contrast (Ph3) and GFP images of representative cells are shown in (A) and (C). Scale bars represent 2 μm on these images. The number of detectable GFP-VsrA foci per cell were analyzed for each cell population. Then, the proportion of cells with minimum one detectable GFP-VsrA focus is indicated next to each GFP image. Minimum 300 cells were analyzed for each biological replicate. (B) and (D) show the percentage of cells displaying a given number of GFP-VsrA foci per cell for each population. The plotted values are averages of at least three independent biological replicates. Error bars correspond to standard deviations (±SD). Statistically significant differences comparing *WT* and *ΔscmA* cells using a Student’s *t* test is indicated as follows: ∗*p* < 0.05.

One of our recent studies successfully used fluorescence microscopy to visualize the MMR process in live *C. crescentus* cells.^49^ It showed that a fluorescently tagged MutL endonuclease forms more stable and distinct fluorescent foci when it initiates the repair of mismatches during the MMR process. Following a similar approach, we therefore constructed *C. crescentus* strains expressing a fluorescently tagged VsrA protein (*gfp-vsrA* replacing the native *vsrA* gene) to try to visualize the putative VSP repair process in live *C. crescentus* cells. Fluorescence microscopy analyses showed that GFP-VsrA did form one or more fluorescent foci in ∼20% of the *WT* cells and in ∼6% of the *ΔscmA* cells cultivated exponentially in PYE (Figures 6A and 6B). These foci appeared at relatively random subcellular positions (Figure 6A) and were not co-localized with the replisome (data not shown), as expected if they are active VSP repair sites located anywhere on the genome. The lower frequency of GFP-VsrA foci in *ΔscmA* compared to *WT* cells may be linked with a lower probability of finding GT mismatches in a YGCCGGCR motif in that strain given that the cytosines are not methylated, although there is not enough evidence to support this hypothesis yet.

Interestingly, previous studies done on *E. coli* cells have shown that the VSP repair process is more active in stationary phase than in exponential phase, either because Vsr levels are higher, or because the accidental conversion of 5mC into T may be more frequent during such stress conditions.^29^^,^^53^ Supporting this model, we also found that GFP-VsrA formed far more fluorescent foci during stationary than exponential phase in *WT* and *ΔscmA* cells with more than 50% of the cells displaying minimum one focus (Figures 6C and 6D). If these foci represent active VSP repair sites, it may indicate that TG mismatches occur at higher frequency in *C. crescentus* cells cultivated in stationary phase and/or that VsrA is more abundant/active during stationary phase.

### MMR proteins are most likely not involved in the putative VsrA-dependent VSP repair process

Not only in *E. coli*, but also in *N. gonorrhoeae*, interesting cross-talks have been proposed to take place between the MMR and the VSP repair processes that can, in principle, both detect and repair TG mismatches in DNA motifs carrying 5mC.^29^^,^^33^

First, it was shown that their MutL protein (bridging MutS and MutH in *E. coli*, while acting as the MMR endonuclease in *N. gonorrhoeae*) interacts directly with their Vsr protein(s), potentially impacting the VSP repair process.^33^^,^^54^^,^^55^ To test whether the *C. crescentus* MMR proteins may interact with the *C. crescentus* VsrA protein, we used bacterial two hybrid assays (BACTH). In brief, we constructed plasmids expressing MutS or MutL proteins fused to the N-terminus of the T18 fragment of the adenylate cyclase and plasmids expressing VsrA fused to the C-terminus of the T25 fragment. These plasmids were then introduced into a BTH101 *E. coli* strain for BACTH assays. Results from these indicate that VsrA can neither interact with MutL, nor with MutS, at least in *E. coli* cells (Figure S11). Thus, if *C. crescentus* MMR proteins have an influence on the VSP repair process, it is probably not through direct interactions between their early actors.

Second, the *E.coli* MutS protein was previously shown to stimulate the VSP repair process *in vivo* and *in vitro*, most likely by helping in the recruitment of Vsr to TG mismatches.^55^ Conversely, another study indicated that the *N. gonorrhoeae* MutS protein reduces the nicking activity of Vsr near TG mismatches *in vitro*, maybe through a competition mechanism during the recognition of TG mismatches.^33^ Then, to test whether the *C. crescentus* MutS protein may affect the capacity of VsrA to potentially cut YGCCGGCR motifs containing TG mismatches on the chromosome of *ΔscmA* cells (Figure 5B), we compared the level of the SOS response in cells expressing or not *scmA* and that lacked MutS (*ΔmutS*) using the P*imuA::lacZ* reporter. We found that the weak SOS response was still induced in response to the loss of ScmA, even in the absence of MutS (1.6-fold induction in the *ΔmutS* background, compared to 1.7-fold induction in the presence of MutS in Figure S7). Thus, MutS does not seem to promote the VsrA-dependent DNA damage response that takes place in *ΔscmA* cells. Supporting this, we also observed that GFP-VsrA formed fluorescent foci at a relatively similar frequency in cells lacking MutS as in cells expressing *mutS* independently of the presence of the ScmA MTase (Figures S12A and S12B) even in cells that were cultivated in stationary phase when these foci are the most visible. Altogether, none of these observations suggest that VsrA may compete or cooperate with MutS in *C. crescentus* cells.

## Discussion

In this study, we used a combination of genetic, genomic, and phenotypic analyses to characterize the role of the ScmA solitary cytosine MTase in *C. crescentus*. We used 5mC-dependent restriction enzymes to confirm a former prediction (Figure 1) stating that ScmA may methylate the first cytosine in the many YGCCGGCR motifs located on the *C. crescentus* chromosome.^35^ A detailed analysis of the phenotypes (Figures 2A, S1, and S2) and fitness (Figure 2B) of *ΔscmA* cells revealed that they were significantly less competitive than *WT* cells when co-cultivated in standard growth conditions. First, hypothesizing that ScmA-dependent methylation may modulate gene expression through epigenetic mechanisms of regulation like several other solitary MTases,^1^^,^^15^^,^^56^ we evaluated the impact of ScmA on the *C. crescentus* transcriptome looking for potential explanations for the observed fitness loss. This analysis revealed that a vast majority of the genes that were affected in *ΔscmA* cells encoded proteins that were known to be involved in the adaptation of *C. crescentus* to DNA damage (Figures 3A and S4, and Table S4). Population-level and single cell reporters were then used to show that an SOS response was indeed turned on (Figure 4A) but only in a subset of *ΔscmA* cells (Figures 4B and 4C). Among the DNA damage-inducible genes^42^ that were significantly activated in *ΔscmA* cells, we identified *vsrA* (Figure 3A and Table S4), encoding a putative Vsr-like endonuclease. Considering that Vsr is functionally linked with Dcm in *E. coli*, we hypothesized that VsrA may similarly be functionally linked with ScmA in *C*. *crescentus*, even if the *vsrA* gene is not genetically linked with the *scmA* gene (these two genes are located at nearly opposite chromosomal positions) unlike the *vsr* gene of *E. coli* that is in the same operon as *dcm*.^31^ We anticipated that this putative endonuclease could potentially play a role in the apparent DNA damage taking place in a subset of *ΔscmA* cells. Indeed, further transcriptome/single cell SOS reporter assays using double *ΔscmA ΔvsrA* mutant cells clearly demonstrated that the SOS response that is turned on in *ΔscmA* cells is strictly dependent on the presence of *vsrA* (Figures 3B and 4, and Table S5). Furthermore, competition assays demonstrated that the loss of fitness associated with the absence of ScmA was also strictly dependent on the presence of *vsrA* (Figure S10). Strikingly, once the *vsrA* gene was removed from *C. crescentus* cells, ScmA had no left-over impact on the expression of genes/operons that appeared to have YGCCGGCR motifs in their promoter region (Table S5). Thus, the ScmA-dependent methylation of the 91 promoter/IG regions containing YGCCGGCR motifs (note that the IG region upstream of the *vsrA* ORF does not include this motif) on the *C. crescentus* genome does not appear to modulate gene expression, at least in the tested growth conditions.

What is then the biological role of ScmA in *C. crescentus*? One option is that cytosine methylation by ScmA may play an epigenetic regulatory role but only when *C. crescentus* cells are cultivated in other growth conditions that have not yet been identified. This would be reminiscent of what happens with Dcm in *E. coli*^18^ and VchM in *V. cholerae*.^21^^,^^22^ ScmA may, alternatively, play a regulatory role in other bacterial strains/species while being only recently acquired by *C. crescentus* from these other bacteria through HGT. Another option is that *scmA* may be a part of an MGE^12^ inducing its own pre-methylation before it moves to other bacterial strains/species by HGT; this would then increase the chances of the MGE to be efficiently transferred into another genome if that genome encodes an RMS that can target the YGCCGGCR motif when it is not methylated. In this case, ScmA would act as a kind of selfish anti-root mean square (RMS) element, promoting the HGT of the MGE. To get insight into this possibility, we used ICEfinder^57^ and PHASTEST^58^ to evaluate whether *scmA* may be included into a prophage or an integrative or conjugative element (ICE) but these tools did not provide evidence that *scmA* may be located within an MGE, even if many genes of unknown function do surround *scmA* on the *C. crescentus* genome (Figure 1A). *scmA* was also not in the prophage identified in the *WT*/NA1000 *C. crescentus* strain that we used in this study.^39^ It is however still possible that *scmA* may be found in an unknown and relatively original type of MGE that is not yet listed/considered by ICEfinder or PHASTEST. A last option that we believe is worth discussing is that the methylation of cytosines by ScmA may play a role in accelerating the speed of evolution at YGCCGGCR motifs in the genomes of bacteria that encode it, since 5mC were previously shown to be C-to-T mutation hot spots in other bacterial species.^26^^,^^28^ Although we did not test whether mutations occur at a higher frequency at YGCCGGCR motifs than elsewhere on the *C. crescentus* genome, we have been recently stunned by a study showing that the closely related *C. crescentus* CB15 strain (GenBank ID: AE005673.1), which is the ancestor of the NA1000 strain that we used in our study (after generations of cultivation in different laboratories around the world), displays only 11 differences compared to NA1000: one consists in a prophage that is integrated into the NA1000 but not into the CB15 genome and the other 10 are single nucleotide polymorphisms (SNPs).^39^ Strikingly, two of these SNPs are C-to-T mutations in YGCCGGCR motifs that are methylated by ScmA in the CB15 and NA1000 strains. Finding that 20% of the SNPs that occurred during this short-term evolution period are specifically located in YGCCGGCR motifs is far more than expected randomly (these motifs represent only 0.6% of the genome). This may just be a coincidence, or it may be seen as first evidence that 5mC bases tend to promote the frequency of C-to-T mutations on the *C. crescentus* genome thereby accelerating its evolution despite the presence of a putative VSP repair system.

Is VsrA a VSP repair protein in *C. crescentus*? Finding a functional link between the ScmA-dependent cytosine methyltransferase and the Vsr-like VsrA protein made us hypothesize that VsrA could be a Vsr protein involved in the VSP repair of TG mismatches arising when 5mC get accidentally deaminated into T in YGCCGGCR motifs methylated by ScmA (Figure 5A). Supporting the proposition that VsrA can be a functional homolog of the well-characterized Vsr^EC^ protein of *E. coli*, we found significant similarities between the predicted 3D structures of Vsr^Ec^ and VsrA using chimeraX 1.8 (Figure S9). In particular, both proteins displayed a four-stranded β-sheet flanked with α-helices on both sides, which is shared by all the members of the PD-(D/E)XK superfamily of nucleases.^50^ Based on this predicted structure, the amino acids located in the nucleolytic active site of Vsr^Ec^ (E25, D51, H64, H69, and D97) could correspond to the E73, D102, D115, H119, and D124 residues of VsrA, suggesting that VsrA may also use magnesium-water clusters to nick DNA motifs as do the three functional Vsr proteins of *E.coli* and *N. gonorrhoeae* do.^32^^,^^51^^,^^52^ The presence of a D115 residue in VsrA instead of the H64 residue in Vsr^Ec^ is reminiscent of what was previously described for the functional Vsr proteins of *N. gonorrhoeae* (V.NgoAXIII)^32^ and *N. meningitidis* (V.Nme18VIP).^34^ Interestingly, *in vitro* experiments showed that these two Vsr proteins can recognize TG mismatches in relatively degenerate DNA motifs that are methylated by any one of the multiple cytosine MTases that are encoded by each of these genomes. Then, we can envision that the *C. crescentus* VsrA may be able to recognize and initiate the repair of TG mismatches located in a variety of DNA contexts. Obviously, more experimental data will be necessary to clarify the exact nucleolytic activity/specificity of VsrA and thereby confirm its role in a VSP repair process in *C. crescentus*. Another interesting outlook of this study will consist of determining whether the GFP-VsrA foci that we distinguished in *C. crescentus* cells (Figure 6) correspond to active VSP repair sites.

Why is *vsrA* expression induced during the SOS response and in (a subset of) *ΔscmA* cells? We initially focused on the *vsrA* gene during this study because our RNA-seq data showed that the levels of the *vsrA* mRNA were significantly induced in *ΔscmA* cells compared to *WT* cells (Figure 3A and Table S4) and because Vsr-like proteins can be functionally linked with solitary cytosine MTases. It was however unlikely that ScmA-dependent DNA methylation could have a direct impact on *vsrA* transcription since the IG region immediately upstream of the *vsrA* ORF does not contain a YGCCGGCR motif methylated by ScmA (Figure S13A). Instead, *vsrA* expression is probably induced as a part of the DNA damage/SOS response that is detected in *ΔscmA* cell populations since *vsrA* is known to be activated during the well-characterized DNA damage-response of *C. crescentus*.^42^ To shed light on how *vsrA* transcription may be activated in *ΔscmA* cells and during DNA damage/SOS responses more generally, we mapped active promoter regions up to 754 bp upstream of the *vsrA* ORF using reporters (Promoter*lacZ* transcriptional fusions shown in Figure S13B) and compared their activities in *WT* and *ΔscmA* cells that were exposed or not to DNA damaging conditions (UV treatment as done in Modell et al.^42^). These analyses revealed that *vsrA* is mostly transcribed from a promoter (named P1 in Figure S13B) located upstream of the *CCNA_02931* ORF that is then apparently in the same operon as *vsrA*. The activity of this promoter is however neither affected by ScmA, nor by DNA damaging conditions (Figures S13C and S13D in M2G and PYE media, respectively); consistently, it does not contain a YGCCGGCR motif or a putative LexA box^41^ (Figure S13A). To understand how the *vsrA* mRNA levels increase in *ΔscmA* cells (Figure 3A and Table S4) and during DNA damaging conditions,^42^ we also looked for putative LexA boxes inside the *vsrA* ORF. This analysis revealed the existence of a GTTC(N7)GTTC direct repeat ∼133 bp after the *vsrA* start codon (Figure S13A), corresponding to the exact known consensus of the *C. crescentus* LexA binding site.^41^ Considering that regulatory LexA binding sites can be found as far as 1 kbp into ORFs in other bacterial species,^59^ we now suspect that it is the elongation of *vsrA* transcription that is repressed by LexA in non-stressed *WT* cells and de-repressed during SOS-inducible conditions (exogenous or endogenous DNA damaging conditions). Consistent with this hypothesis, it is worth mentioning that the 3′ end of a small sense RNA (named CCNA_R0176 and shown in Figure S13A) has been mapped very close to this LexA box when sequencing whole RNA samples from *WT* cells^60^; it could correspond to the 3′ end of a truncated CCNA_2931-vsrA multicistronic mRNA when LexA represses *vsrA* but not *CCNA_02931* transcription in unstressed cells. Independently of the precise mechanism of activation that is in place, promoting the expression of *vsrA* during the SOS response appears as a potentially useful process since it is known that several error-prone DNA polymerases are activated during the SOS response in *C. crescentus*^43^; VsrA may then contribute to detecting and repairing base mismatches that appear more frequently during the SOS response than in unstressed cells.

Why is VsrA inducing an SOS response in a subset of *ΔscmA* cells? The answer to this question remains elusive but one option is that TG mismatches created by the DNA Pol III during the replication process, and that are not detected on-time by its proofreading activity, can not only be recognized by MutS^49^ but also by even basal levels of VsrA, at least when located in YGCCGGCR DNA motifs (Figure 5B). Several studies tried to estimate the spontaneous mutation rate of *C. crescentus* cells by measuring the spontaneous appearance of rifampicin-resistant mutants in *WT* populations; these can appear if specific base substitutions take place in a very restricted region of the *rpoD* gene.^36^^,^^43^^,^^49^^,^^61^ Using such assays, it was found that rifampicin-resistant mutants appeared at a frequency between 10^−7^ and 10^−8^ in *WT* populations despite an efficient MMR process and the very low expression of error-prone DNA polymerases during optimum growth conditions. Then, considering that *C. crescentus* has more than 3,000 YGCCGGCR motifs on its genome, TG mismatches in such motifs may be found in ∼1 out of 10^4^–10^5^ unstressed *ΔscmA* cells, or at even higher frequencies in stressed *ΔscmA* cells with an SOS response where error-prone DNA polymerases like ImuABC are induced.^43^ In these cases, VsrA may cut the two non-methylated DNA strands (Figure 5B), instead of just nicking the only DNA strand that is not methylated as is the case in *WT* cells (Figure 5A), leading to the formation of accidental DSB and to a transient DNA damage response to promote the repair of this DSB. This hypothesis is reminiscent of what happens in *E. coli dam* mutant cells, where basal levels of the MutH endonuclease accidentally creates DSB instead of nicking a single DNA strand when trying to repair mismatched bases during the MMR process.^45^^,^^48^ Since *vsrA* transcription was found to be activated in *ΔscmA* cells, coincident with the SOS response that was detected in a subset of cells, we wondered if an over-production of VsrA may be sufficient to induce or to promote endogenous DSBs/DNA damage in *C. crescentus* cells. To test this, we constructed a medium-copy number plasmid over-expressing *vsrA* from a xylose-inducible promoter (pBX-VsrA) and introduced it in *WT* and *ΔscmA* cells containing the P*imuA::lacZ* SOS reporter. Follow-up β-galactosidase assays using cells cultivated in the presence of the xylose inducer showed that the artificial over-expression of *vsrA* was neither sufficient to activate an SOS response in *WT* cells, nor to exacerbate the SOS response that can be detected in *ΔscmA* cell populations (Figure S14). Overall, this result suggests that basal levels of VsrA may not be limiting to accidentally create DSBs in rare *ΔscmA* cells with TG mismatches that need to be repaired, even if the SOS response that is subsequently turned on in these cells will generate a positive feedback loop boosting VsrA levels (Figure 5B).

A surprising finding during this study was that we discovered a functional link between a cytosine MTase and a Vsr-like protein that are however not encoded by genes that belong to the same operon and that are not even genetically linked on the *C. crescentus* chromosome. This contrast with the Dcm/Vsr^Ec^ pair found in *E. coli*^31^ but, to some extent, shows similarities with a few *Neisseria* Vsr proteins that can repair TG mismatches in a diversity of DNA contexts.^34^ Still, in *Neisseria*, the genes encoding these characterized Vsr proteins were systematically found next to a MTase gene (that was sometimes truncated), unlike what we found in *C. crescentus*. Such distancing between Vsr- and MTase-encoding genes on bacterial genomes may then promote their horizontal transfer as “solitary” elements and could potentially explain why “solitary” Vsr-encoding genes have now been observed in sequenced bacterial genomes.^38^ Our study also suggests that the presence of a *vsr*-like gene can stabilize a cytosine MTase-encoding gene in a bacterial genome since we showed that the presence/expression of *vsrA* seriously compromised the fitness/competitiveness of *C. crescentus* cells that lost their *scmA* gene (Figure 2B compared to Figure S10). Altogether, our study paves the way to better understanding the impact, the transmission and the stabilization of solitary cytosine MTases in a variety of bacteria.

### Limitations of the study

While this study provide evidence that ScmA methylates cytosines in YGCCGGCR motifs, we did not find an obvious biological role for this solitary cytosine MTase in *C. crescentus* cells cultivated under standard growth conditions. Future studies should now focus on searching for a role in a variety of growth conditions or beyond *C. crescentus*. Another limitation is that we have not yet characterized the exact molecular activity of VsrA to prove that it can detect and repair TG mismatches located in the many YGCCGGCR motifs methylated by ScmA on the *C. crescentus* genome. This will be important to then examine at which subcellular location, and at which frequency, the VSP repair process takes place in *C. crescentus*.

## Resource availability

### Lead contact

Further information and requests for resources and reagents should be directed to and will be fulfilled by the lead contact, Justine Collier (justine.collier@unil.ch).

### Materials availability

Plasmids and strains generated in this study are stored in the lead contact laboratory and are available upon request. This study did not generate unique reagents.

### Data and code availability

• RNA-seq data are available as NCBI GEO (GSE286545 and GSE286547 with links in the key resources table).
• This study did not involve unique codes.
• This study did not involve other unique items.

## Acknowledgments

We thank Tiancong Chai and Jacqueline Masternak for their technical contribution during the construction of strains/plasmids. We also thank Pedro Oliveira, Laurent Casini, Sandra Martin, Florian Fournes, and Corentin Jaboulay for helpful discussions during the project. We finally thank other members of the Lausanne Genomic Technologies Facility for RNA-seq library preparations and sequencing.

## Author contributions

N.M., G.W., and J.C. designed the research; N.M., G.W., N.P., and J.C. planned the experiments; N.M., G.W., N.P., K.B., and J.M. performed and/or analyzed experiments; N.M. and J.C. wrote the article. All the authors edited the article.

## Declaration of interests

The authors declare no competing interests.

## STAR★Methods

### Key resources table

**Table:** 

REAGENT or RESOURCE	SOURCE	IDENTIFIER
**Deposited data**		

RNAseq data related to Table S4	NCBI GEO	GSE286545: https://www.ncbi.nlm.nih.gov/geo/query/acc.cgi?acc=GSE286545
RNAseq data related to Table S5	NCBI GEO	GSE286547: https://www.ncbi.nlm.nih.gov/geo/query/acc.cgi?acc=GSE286547

**Experimental models: Organisms/strains**		

Listed with references in Table S1	–	–

**Oligonucleotides**		

Listed in Table S3	Sigma-Aldrich	–

**Recombinant DNA**		

Listed with references in Table S2	–	–

### Experimental models and study participant details

Bacterial strains used in this study are listed in Table S1 available in supplemental information.

### Method details

#### Growth conditions

*E. coli* cells were cultivated in/on Luria-Bertani (LB) medium or on LB agar (1.5%) plates at 37°C. *C. crescentus* strains were grown in/on Peptone Yeast Extract (PYE+/-1.5% agar) complex medium or in M2G minimal medium.^62^ When required to maintain plasmids into strains, antibiotics were added into the medium at the following concentrations (liquid/plates): kanamycin 30/50 μg/ml, streptomycin 30/30 μg/ml, gentamycin 15/20 μg/ml, oxytetracycline 12/12 mg/ml and ampicillin 50/100 mg/ml for *E. coli*; kanamycin 5/25 μg/ml, spectinomycin 25/100 μg/ml, gentamycin 1/5 μg/ml and oxytetracycline 1/1 μg/ml for *C. crescentus*. During BACTH assays, isopropyl β-d-1-thiogalactopyranoside (IPTG, AppliChem) was added at a final concentration of 0.5 mM and 5-bromo-4-chloro-3-indolyl-β-d-galactopyranoside (X-gal, AppliChem) was added at a final concentration of 40 μg/ml to detect β-galactosidase (LacZ) activity on plates.

#### Molecular cloning procedures and plasmids constructions

DNA manipulations and molecular cloning procedures were performed according to standard protocols. All constructs were verified by colony PCR and/or Sanger sequencing (Microsynth AG). Plasmids and oligonucleotides used in this study are listed in Tables S2 and S3, respectively, available in supplemental information.

##### Derivative of pBXMCS-2

To construct pBX-1motif: a first 878 bp sequence containing a 5′-end HpaII motif and a 3′-end YGCCGGCR motif and a second 1359 bp sequence containing a 3′-end HpaII motif, were both amplified using primer pairs NM210/NM211 or NM212/213 using chromosomal *C. crescentus* NA1000 gDNA as a template. The two fragments were digested with HindIII/KpnI and KpnI/SpeI, respectively, and cloned into the HindIII/SpeI-digested pBXMCS-2 vector by triple ligation.

To construct pBX-VsrA: the *vsrA* ORF was amplified with primer pair NM91/NM92 using chromosomal *C. crescentus* NA1000 gDNA as a template. The fragment was subsequently digested with NdeI/EcoRI and ligated into the NdeI/EcoRI -digested pBXMCS-2 vector.

##### Derivatives of pNPTS138

• To construct pNPTS138*::ΔscmA*: the ∼500 bp sequences upstream and downstream of the *scmA* (*CCNA_01085*) ORF were amplified with primer pairs TC79/TC80 or TC81/TC82 using chromosomal *C. crescentus* NA1000 gDNA as a template. The two fragments were digested with SpeI/BamHI and BamHI/EcoRI, respectively, and cloned into the SpeI/EcoRI-digested pNPTS138 vector by triple ligation.
• To construct pNPTS138*::ΔvsrA*: the ∼500 bp sequences upstream and downstream of *vsrA* (*CCNA_02930*) ORF were amplified with primer pairs NM31/NM32 or NM33/NM34 using chromosomal *C. crescentus* NA1000 gDNA as a template. The two fragments contained 33-nucleotide overhangs and were used as templates for overlap extension PCR in a second round of PCR using the primer pair NM31/NM34. The fragment was subsequently digested with SpeI/BamHI and cloned into the SpeI/BamHI-digested pNPTS138 vector by double ligation.
• To construct pNPTS138*::Δ2876*: the ∼500 bp sequences upstream and downstream of *CCNA_02876* were amplified with primer pairs NM27/NM28 or NM29/NM30 using chromosomal *C. crescentus* NA1000 gDNA as a template. The two fragments contained a 34-nucleotide overhang and were used as templates for overlap extension PCR in a second round of PCR using primer pair NM27/NM30. The fragment was subsequently digested with SpeI/BamHI and cloned into the SpeI/BamHI-digested pNPTS138 vector by double ligation.
• To construct pNPTS138::P*imuA::mCherry*: the ∼500 bp sequences upstream and downstream of the 3′-end of *imuA* were amplified with primer pairs NM218/NM219 or NM222/NM223 using chromosomal *C. crescentus* NA1000 gDNA as a template. The *mCherry* ORF was amplified from pCHYC-1 using the primer pair NM220/NM221 that created a 5′-aaagaggagaaa-3′ ribosome binding site located in frame with the *mCherry* ORF. The three fragments containing 26-29-nucleotide overhangs were then used as templates for overlap extension PCR in a second round of PCR using the primer pair NM218/NM223. The fragment was subsequently digested with SpeI/EcoRI and cloned into the SpeI/EcoRI-digested pNPTS138 vector by double ligation.
• To construct pNPTS138::P*bapE::mCherry*: the ∼500 bp sequences upstream and downstream of the 3′-end of *bapE* were amplified with primer pairs NM114/NM115 or NM118/NM119 using chromosomal *C. crescentus* NA1000 gDNA as a template. The *mCherry* ORF was amplified from pCHYC-1 using the primer pair NM116/NM117 that created a 5′-aaagaggagaaa-3′ ribosome binding site in frame with the *mCherry* ORF. The three fragments containing a 26-29-nucleotide overhangs were used as templates for overlap extension PCR in a second round of PCR using primer pair NM114/NM119. The fragment was subsequently digested with SpeI/EcoRI and cloned into the SpeI/EcoRI-digested pNPTS138 vector by double ligation.
• To construct pNPTS138::*GFP::vsrA*: the ∼500 bp sequences upstream and downstream of the 5′ end of the *vsrA* ORF were amplified with primer pairs NM169/NM170 and NM173/NM174 using chromosomal *C. crescentus* NA1000 gDNA as a template. The *GFP* gene without its stop codon was amplified from pGFPC-1 using the primer pair NM171/NM172. The three fragments containing 22-30-nucleotide overhangs were used as templates for overlap extension PCR in a second round of PCR using the primer pair NM169/NM174. The fragment was subsequently digested with SpeI/EcoRI and cloned into the SpeI/EcoRI-digested digested pNPTS138 vector by double ligation.

##### Derivatives of pUT18

• To construct pUT18::*mutS*: the *mutS* ORF was amplified with primer pair NM201/NM202 using chromosomal *C. crescentus* NA1000 gDNA as a template. The fragment was subsequently digested with BamHI/KpnI and cloned into the BamHI/KpnI-digested pUT18 BACTH vector by double ligation.
• To construct pUT18::*mutL*: the *mutL* ORF was amplified with primer pair NM203/NM204 using chromosomal *C. crescentus* NA1000 gDNA as a template. The fragment was subsequently digested with BamHI/KpnI and cloned into the BamHI/KpnI-digested pUT18 BACTH vector by double ligation.

##### Derivative of pKT25

To construct pKT25::*vsrA*: the *vsrA* ORF was amplified with primer pair NM205/NM206 using chromosomal *C. crescentus* NA1000 gDNA as a template. The fragment was subsequently digested with BamHI/KpnI and cloned into the BamHI/KpnI-digested pKT25 BACTH vector by double ligation.

##### Derivatives of p*lacZ*290

To construct pP1+P2::*lacZ290:* the 754 bp region upstream of the *vsrA* ORF was amplified with primer pair NPJC30/NPJC32 using chromosomal *C. crescentus* NA1000 gDNA as a template. The fragment was subsequently digested with HindIII/EcoRI and ligated into the HindIII/EcoRI -digested p*lacZ*290 vector.

To construct pP2::*lacZ290:* the 452 bp region upstream of the *vsrA* ORF was amplified with primer pair NPJC30/NPJC31 using chromosomal *C. crescentus* NA1000 gDNA as a template. The fragment was subsequently digested with HindIII/EcoRI and ligated into the HindIII/EcoRI -digested p*lacZ*290 vector.

#### Strain constructions

• Integrative or replicative plasmids were introduced into *C. crescentus* cells by electro-transformation.
• Gene deletions or gene replacements on the *C. crescentus* chromosome were done using pNPTS138 derivatives and a two-step recombination procedure. Briefly, pNPTS138 derivatives were first introduced into the chromosome of *C. crescentus* cells by transformation and a first event of homologous recombination at the targeted locus (selection on PYEA+Km plates). A few colonies were then cultivated over-night in PYE (no Km; plasmid re-excision step via a second homologous recombination event) and subsequently plated on PYEA+Sucrose 3% to select the colonies that lost the *sacB* gene/plasmid. Sucrose-resistant and Km-sensitive colonies were then screened and the genotype of these colonies were differentiated by colony-PCR (one expects to find ∼50% of *WT* and ∼50% of genetically-modified colonies at that step). This method was used to construct the following strains: JC2005, JC2540, JC2542, JC2987, JC2474, JC2539, JC2541, JC3104, JC3105, JC3107, JC3109, JC2813, JC2814, JC2815, JC2860, JC2861, JC3072 and JC3075.
• To construct the JC2985, JC2984 and JC3049 strains, the pVGFPC-1 and pVGFPC-4 integrative plasmids were inserted at the native *vanA* locus on the *C. crescentus* chromosome by homologous recombination, which was confirmed by PCR.^63^

#### Restriction enzyme-based detection of 5mC bases

The pBX-1motif plasmid was isolated from overnight cultures of *C. crescentus* cells cultivated in PYE medium or of *E. coli* cells cultivated in LB medium. 700 ng of pBX-1motif were digested for 2h at 37°C using the 5mC-sensitive HpaII or MspI restriction endonucleases (New England Biolabs, USA). The size of the resulting restriction fragments was then estimated using agarose gel electrophoresis.

#### Fitness assays comparing mono- and co-cultures of *C. crescentus* strains

*C. crescentus* strains were cultivated overnight in PYE medium. Stationary phase mono-cultures were then diluted into 3mL of PYE to reach a starting OD_660nm_ of ∼0.05. For co-cultures to compare the fitness of two strains, an approximately equal numbers of cells of each genotype was inoculated simultaneously into 3mL of PYE to reach a starting OD_660nm_ of ∼0.05 (= OD_660nm_ of ∼0.025 for each strain). Mono- and co-cultures were then diluted back 1000-fold (still in PYE) after 7 hours of growth and again 20-fold after 17 more hours of growth to maintain cells mostly in exponential phase for 24 hours. These 24 hour-cycles were repeated for a total duration of 5 days (Figures 2 and S10) or 4 days (Figure S3). The doubling time (dt) of each culture was estimated using the following formula: dt = ln2/r, with r corresponding to the growth rate. We thereby estimated that cells went through ∼51 generations during this 5-day period (or ∼40 generations during the 4-day period). At regular time intervals (before each dilution), aliquots of mono- or co-cultures were collected and serially diluted prior to plating onto PYEA+Spec and PYEA+Gent to count colony-forming units per mL of culture (CFU/mL) corresponding to each genotype.

#### Microscopy and image analysis

Cells were imaged immediately from fresh cultures or after being stored at 4°C for up to a few days after being fixed using a 5X-fix solution (150 mM NaPO4, 12.5% formaldehyde at pH 7.5). Fresh or fixed cells were transferred onto M2 medium with 1% agarose pads on glass slides and were then covered with a coverslip. For phase contrast and fluorescence microscopy, two microscope systems were used depending on the experiments: one (used for Figures 4, S2, and S5) was an AxioImager M1 microscope (Zeiss) as described in^64^ and the other one (used for Figures 6 and S12) was a Leica DMi8 as described in.^65^ The Fiji (imageJ) 2.3.0 software with the MicrobeJ plugin^66^ was used to analyze images to estimate the length of cells or to identify the proportion of fluorescent cells (from SOS reporters) or of cells with a given number of GFP-VsrA foci in a given cell population.

#### RNA extraction

RNA samples were prepared from 10 mL of exponentially growing *C. crescentus* cells cultivated in M2G medium (OD_660nm_∼0.4). Cells were pelleted and immediately frozen in liquid nitrogen prior to storage at -80°C. RNA were extracted using the RNeasy Mini-Kit from Qiagen following the manufacturer’s protocol including a DNase I (RNase-free DNases set from Qiagen) treatment and a second TURBO DNA-free^TM^ DNase treatment (from Invitrogen) following the protocol of the manufacturer. RNA samples were then re-purified using a RNA clean-up procedure (RNeasy Mini-Kit). Absence of DNA contaminations was verified by standard PCR. The quality of RNA samples was verified on an agarose gel and using a Fragment Analyzer (Agilent Technologies): the RNAs had RQNs between 9.7 and 10.

#### RNA-sequencing and analyses

##### RNA-seq library preparation

For Figure 3A and Table S4, RNA-seq libraries were prepared from 1000 ng of total RNA with the Illumina Truseq stranded mRNA Prep reagents (Illumina) using a unique dual indexing strategy and following the official protocol. The polyA selection step was replaced by rRNA depletion step with the Ribo-off rRNA Depletion Kit (Bacteria) (Vazyme). For Figure 3B and Table S5, RNA-seq libraries were prepared from 100 ng of total RNA with the Illumina Stranded mRNA Prep reagents (Illumina) using unique dual indexing strategy, and following the official protocol automated on the Sciclone liquid handling robot (PerkinElmer). The polyA selection step was replaced by rRNA depletion step with the RiboCop for Bacteria mixed bacterial samples reagents (Lexogen). All libraries were quantified by a fluorometric method (QubIT, Life Technologies) and their quality assessed on the Fragment Analyzer. Sequencing was performed either on the Illumina HiSeq 4000 or on the NovaSeq 6000 v1.5 flow cell with single read settings. Sequencing data were demultiplexed using the bcl2fastq2 Conversion Software (version 2.20, Illumina).

##### RNA-seq data processing and analyses

Purity-filtered reads were adapted and quality trimmed with Cutadapt (v. 1.8 for Figure 3A and Table S4 and v. 2.5 for Figure 3B and Table S5).^67^ Upon removal of reads matching to ribosomal RNA sequences (fastq_screen v. 0.11.1) and further filtering for low complexity with reaper (v. 15–065,^68^ reads were aligned against *Caulobacter crescentus na1000.ASM2200v1* genome using STAR (v. 2.5.3a.^69^ The number of read counts per gene locus was summarized with htseq-count (v. 0.9.1;^70^) using *Caulobacter crescentus na1000.ASM2200v1* gene annotation. Quality of the RNA-seq data alignment was assessed using RSeQC (v. 2.3.7;^71^). The counts per gene table was used for statistical analysis. Quality control analysis was performed through sample density distribution plots, hierarchical clustering and sample PCA. For Figure 3A and Table S4, statistical analyses were performed in R (R version 3.6.1). Genes with no counts were filtered out from the dataset. Library sizes were scaled using TMM normalization (EdgeR package version 3.28.1;^72^) and log2-transformed with the limma cpm function (prior counts set to 1). Differential expression was computed with limma (Limma v3.42.2;^73^) by fitting the samples into a linear model using as factors three experimental conditions and the batch and then performing the *ΔscmA* vs *WT* comparison. For Figure 3B and Table S5, statistical analyses were performed in R (R version 4.2.1). Genes with at least 1 count per million (cpm) in at least 3 samples were retained for the analysis. Library sizes were scaled using TMM normalization (EdgeR v3.40.1) and log2-transformed with the limma cpm function (prior counts set to 3). Differential expression was computed with limma (Limma v3.54.0) by fitting the samples into a linear model and performing the *ΔscmA ΔvsrA* vs *ΔvsrA* comparison. For both analyses, moderated t-tests were used for the comparisons and P-value adjustments were done using the Benjamini-Hochberg method.

#### β-galactosidase assays

*C. crescentus* cells were grown exponentially in the indicated media. 200μl of cultures were used for each β-galactosidase assay following a standard protocol.^74^

#### Bacterial adenylate cyclase two-hybrid (BACTH) assays

*E. coli* BTH101 cells were co-transformed with the pUT18 and pKT25 plasmids or with their derivatives by electroporation. ∼50 co-transformants were pooled together and resuspended in 100 μl of LB. 5 μl of this cell suspension was then spotted onto LBA plates containing X-Gal+IPTG+kanamycin+ampicillin and incubated for 48 hours at 30°C. Blue patches then indicate that the two tested proteins interact.

Quantitative β-galactosidase assays were then performed from these bacterial patches as described in.^75^ Briefly, the bacterial patches were collected and resuspended in 1 ml of sterile phosphate-buffered saline (PBS). The suspensions were then diluted 10-fold and 500 μl of this diluted cell suspension was used to perform a β-galactosidase assay as described in.^74^

### Quantification and statistical analysis

Sample sizes (n) are indicated in figure legends. Statistical analyses were carried out using RStudio (version 2024.04.2+764). Statistical analyses were done using two-tailed Student’s *t*-tests.

## Supplemental information

Document S1. Figures S1–S14 and Tables S1–S3

Table S4. RNA-seq results comparing the transcriptome of *ΔscmA* (JC2005) and *WT* (JC450) cells, related to Figure 3A The complete data and a list of genes that were significantly misregulated (adjusted *p* value < 0.05 and log_2_FC ≥ 1 or log_2_FC ≤ −1) are included in two distinct sheets. Significantly misregulated genes are highlighted in gray. On the sheet containing the list of significant results, genes previously identified as DNA-damage induced genes 40, 42 are highlighted in yellow and the presence or absence of a LexA binding site is stated (SOS response genes). The eventual presence of YGCCGGCR motifs in the 250 bp sequences upstream of the annotated start codon of each gene is also indicated.

Table S5. RNA-seq results comparing the transcriptome of *ΔscmA ΔvsrA* (JC2542) and *ΔvsrA* (JC2540) cells, related to Figure 3B The complete data and a list of genes that were significantly misregulated (adjusted *p* value < 0.05 and log_2_FC ≥ 1 or log_2_FC ≤ −1) are included in two distinct sheets. Significantly misregulated genes are highlighted in gray. The eventual presence of YGCCGGCR motifs in the 250 bp sequences upstream of the annotated start codon of each gene is also indicated.

Data S1. Interactive volcano plot corresponding to RNA-seq results comparing the transcriptome of *ΔscmA ΔvsrA* (JC2542) and *ΔvsrA* (JC2540) cells, related to Figure 3B Black and red dots correspond to genes that were significantly misregulated (adjusted *p* value < 0.05 and log_2_FC ≥ 1 in red for “upReg” or log_2_FC ≤ −1 in black for “downReg”); gray dots correspond to genes that were not significantly misregulated (“nonDE”: adjusted *p* value > 0.05 or −1<log_2_FC < 1).

Data S2. Interactive volcano plot corresponding to RNA-seq results comparing the transcriptome of *ΔscmA* (JC2005) and *WT* (JC450) cells, related to Figure 3A Black and red dots correspond to genes that were significantly misregulated (adjusted *p* value < 0.05 and log_2_FC ≥ 1 in red for “upReg” or log_2_FC ≤ −1 in black for “downReg”); gray dots correspond to genes that were not significantly misregulated (“nonDE”: adjusted *p* value > 0.05 or −1<log_2_FC < 1).
