# Supplementary material for: Cytosine methylation contributes to the fitness of Caulobacter cells naturally expressing a Vsr-like protein
Source: iScience. 2026 Jan 20;29(2):114749. doi: 10.1016/j.isci.2026.114749 (PMC12907096; doi:10.1016/j.isci.2026.114749)
Supplement: Document S1. Figures S1–S14 and Tables S1–S3 [file mmc1.pdf]

iScience, Volume *29*

Supplemental information

Cytosine methylation contributes to the fitness of *Caulobacter* cells naturally expressing a Vsr-like protein

Noémie Matthey, Giorgia Wennubst, Nicolas Pellaton, Karolina Bojkowska, Julien Marquis, and Justine Collier

Cytosine methylation by ScmA contributes to the fitness of

***Caulobacter crescentus* cells naturally expressing a Vsr-like protein**

Noémie MATTHEY, Giorgia WENNUBST, Nicolas PELLATON, Karolina BOJKOWSKA,

Julien MARQUIS, and Justine COLLIER

SUPPLEMENTARY DATA AND TABLES

Content:

Pages 2-15: Supplementary Figures S1 to S14 with legends

Pages 16-20: Supplementary Tables S1 to S3 with captions

Page 21: Supplementary References

Supplementary Figures with their legends:

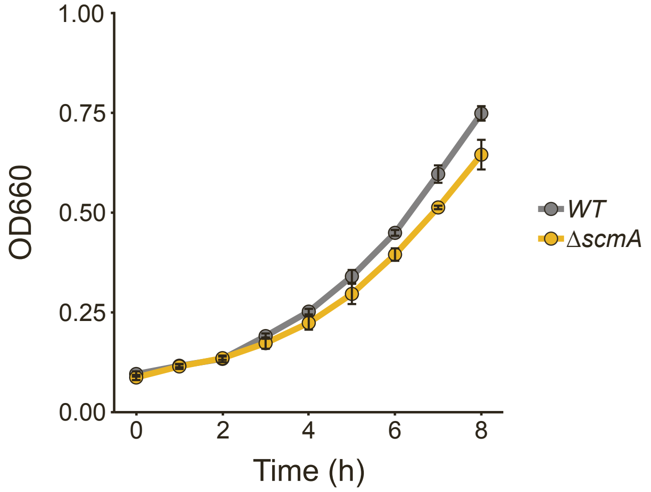

Figure S1: The growth rates of wild-type and Δ*scmA* *C. crescentus* cells cultivated in minimal medium are globally comparable. Growth curves of *WT* (JC450) and *ΔscmA* (JC2005) cells cultivated in minimal M2G medium. Cells were first cultivated overnight in PYE medium (reaching stationary phase) and cultures were then diluted 10-fold into M2G medium. Cells were then cultivated until they reached stationary phase, and the cultures were diluted again into fresh M2G medium to reach an OD_{660nm}~0.1 at time 0. The OD_{660nm} was then measured every hour for 8 hours. The values plotted in these growth curves correspond to the average measurements from at least three independent biological experiments (error bars = ± SD).

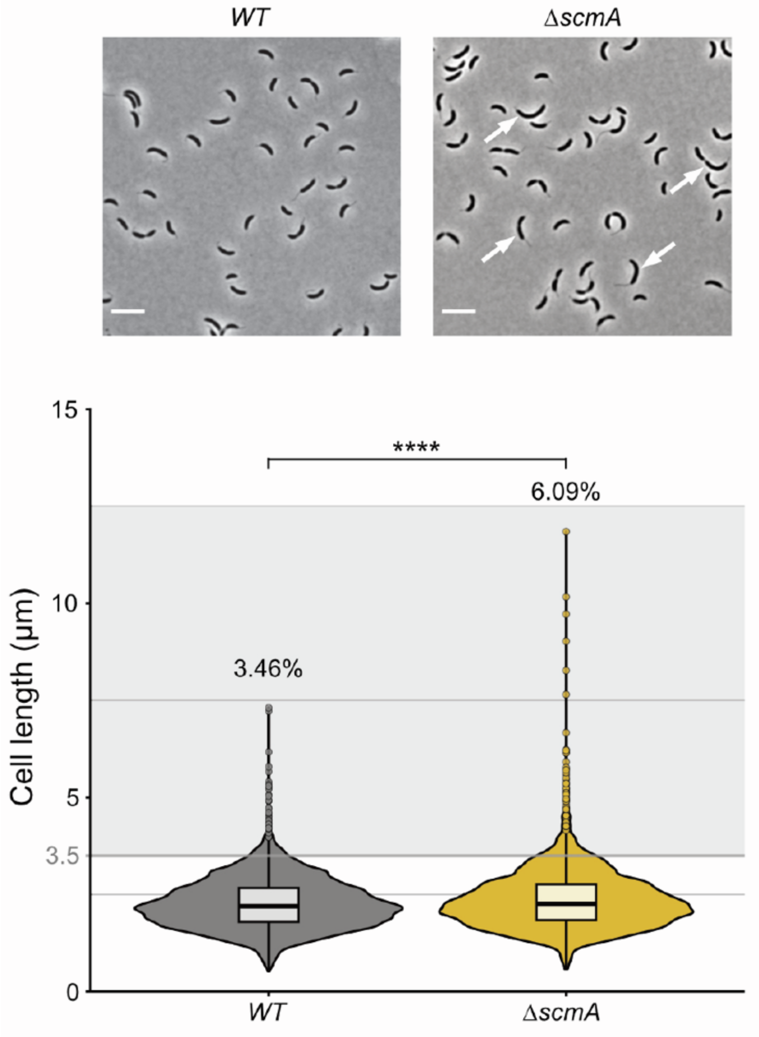

Figure S2: Δ*scmA* cells are more often significantly more elongated than isogenic wild-type *C. crescentus* cells. *WT* (JC450) and Δ*scmA* (JC2005) cells were cultivated exponentially in M2G medium until they reached an OD_{660nm}~0.4. Cells were then fixed before being imaged by phase-contrast microscopy. Representative images are shown in the upper panel with white arrows pointing at significantly elongated cells. Scale bar = 2 µm. The lower panel corresponds to violin plots showing the cell length distribution in each cell population. The length of minimum 900 cells per biological replicate was measured and the values of three independent biological replicates were used to make these violin plots. Cells greater than 3.5 µm-long (grey zone) were considered as significantly elongated compared to the rest of the population; the percentage of these significantly elongated cells in each population is indicated above each plot. The boxes indicate the interquartile range with the center representing the median. Statistical significance is indicated (****=$p < 0.0001$).

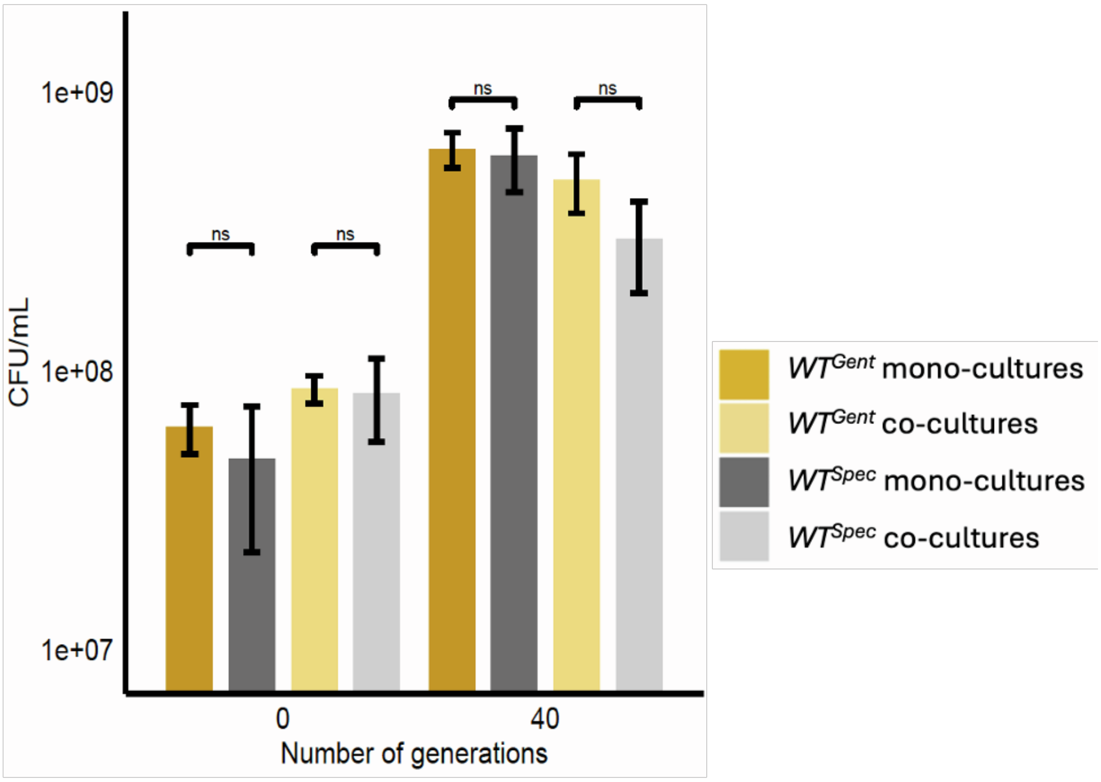

Figure S3: The *Spec* and *Gent* resistance cassettes do not impact the fitness of *WT C. crescentus* cells (control experiment related to Fig.2B). Monocultures and co-cultures of *WT*^{Spec} (JC2985) and/or *WT*^{Gent} (JC2983) cells were cultivated in complex PYE medium for ~40 generations (the generation time of these strains is close to 2 hours under these conditions). Before each regular dilution, spectinomycin-resistant (*WT*^{Spec}) and gentamycin-resistant (*WT*^{Gent}) colony forming units (CFU) per mL of (co-)cultures were measured on antibiotic-containing PYEA plates. The plotted values correspond to average measurements from three independent biological replicates. Error bars correspond to standard deviations (± SD). Statistical significance is indicated (ns = $p > 0.05$).

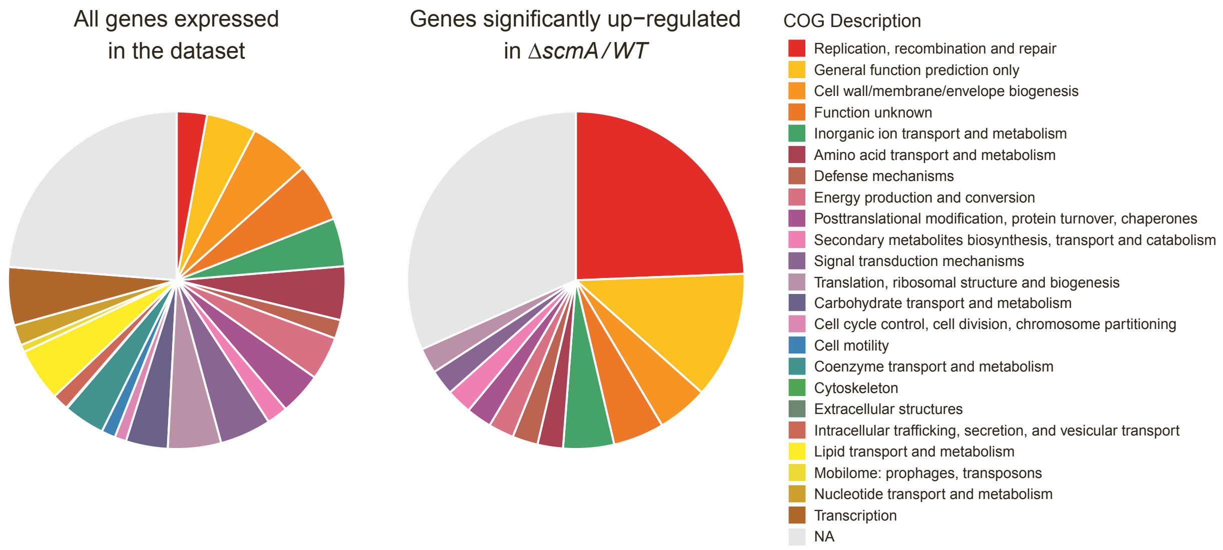

Figure S4: The COG category "Replication, recombination and repair" is particularly over-represented among the genes that are significantly up-regulated in *ΔscmA* compared to *WT* cells. Pie charts representing the proportion of genes in each COG category among all the genes that were expressed during these experiments (same experiments as shown in Fig.3) and among the genes that were significantly up-regulated (adjusted *P*-value >0.05 and/or FC<2) in Δ*scmA* (JC2005) cells compared to *WT* (JC450) cells. A distinct color was assigned to each COG category. NA = no COG assigned. COG categories were assigned to *C. crescentus* genes using the *cogclassifier* tool (v1.0.5) from bioconda. Out of 3'859 UniProtKB entries for the NA1000 strain, 81.58% (3148) protein sequences were classified to a COG functional category.

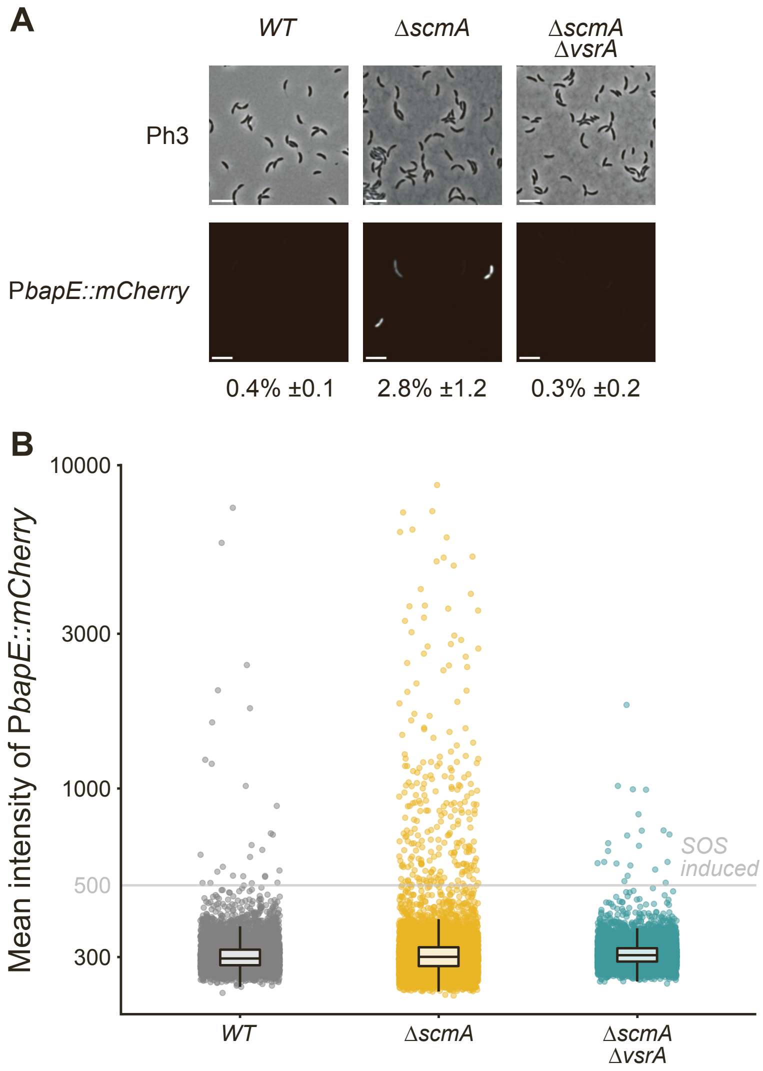

Figure S5: Single-cell fluorescence microscopy assays showing that a VsrA-dependent SOS response is turned on in a subset of *ΔscmA* cells in clonal populations. The P*bapE::mCherry* reporter was integrated at the *bapE* locus on the genome of *WT* (giving JC2813), *ΔscmA* (giving JC2814) or *ΔscmA ΔvsrA* (giving JC2815) cells. The resulting strains were cultivated in exponential phase in PYE complex medium. **(A)** Phase contrast (Ph3) and mCherry images of representative cells are shown. Scale bars indicate 2µm. **(B)** Quantification of the cytoplasmic fluorescence intensity (arbitrary units) of cells in populations from (A). The average cytoplasmic fluorescence intensity of minimum 6000 cells of each strain/culture (3 biological replicates of each with minimum 2000 cells/replicate) is shown: the boxes indicate the interquartile range with the center representing the median. Dots above the "SOS" threshold represented by the grey line were used to estimate the percentage of cells in each population displaying an obvious SOS response, as indicated under each microscopy image in panel (A).

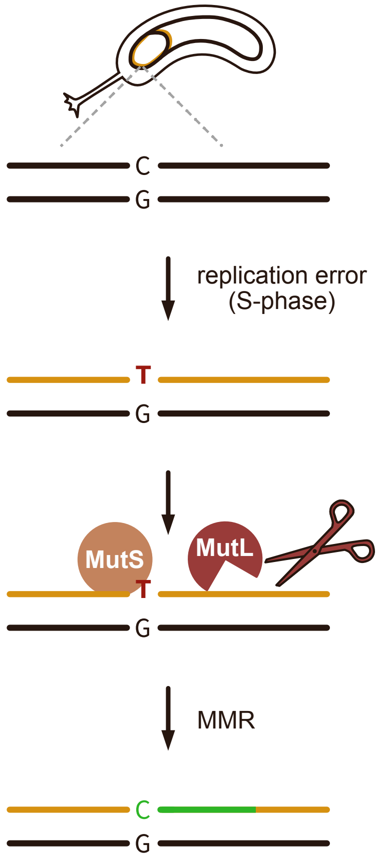

Figure S6: Model for the DNA mismatch repair (MMR) process in *C. crescentus* (inspired from [1]). During DNA replication (S-phase), the DNA Pol III can accidentally mis-incorporate bases in the newly synthesized DNA strand (represented in orange; a TG mismatch is shown as an example). MutS will recognize these mismatches and recruit/activate the MutL endonuclease to nick the newly replicated DNA strand to initiate the repair of the mismatch. The DNA PolIII then re-synthetizes a patch of that DNA strand (shown in green). How MutL recognizes the newly synthetized DNA strand that it needs to nick during this process remains unclear.

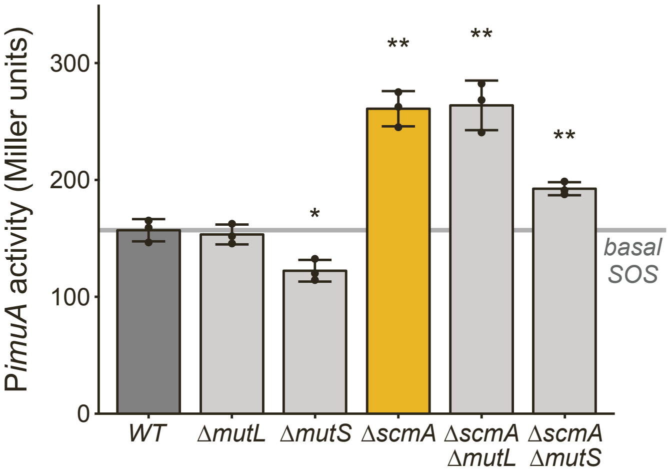

Figure S7: The SOS response that is turned on in a subset of Δ*scmA* cells does not involve MutL or MutS. The P*imuA*::*lacZ*290 reporter was introduced into the chromosome of the indicated *C. crescentus* strains and β-galactosidase assays were performed on cells cultivated exponentially in PYE medium (cultures with an OD_{660nm} ~ 0.3). The grey line represents the average basal SOS response found in *WT* cells. The plotted values correspond to the average promoter activities measured from three independent biological replicates with two technical replicates each (error bars = ± SD). Statistical significance is indicated (*=$p < 0.05$; ** =$p < 0.01$).

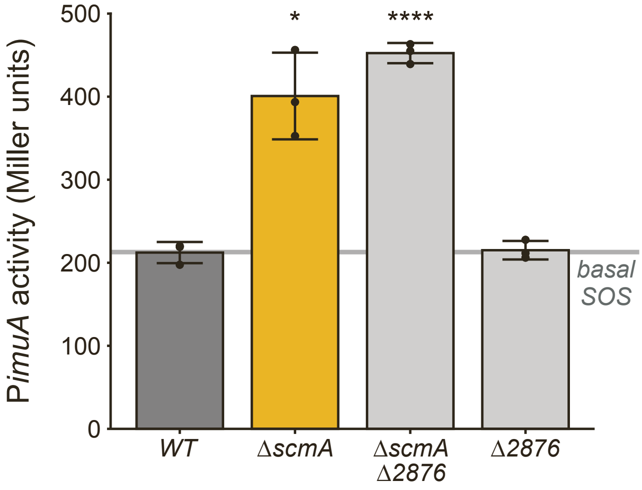

Figure S8: The CCNA_02876 Vsr-like endonuclease is not involved in the SOS response detected in Δ*scmA* cells. The P*imuA*::*lacZ*290 reporter was introduced into the chromosome of the indicated *C. crescentus* strains and β-galactosidase assays were performed on cells cultivated exponentially in PYE medium (cultures with an OD_{660nm} ~ 0.4). The grey line represents the average basal SOS response found in *WT* cells. The plotted values correspond to the average promoter activities measured from three independent biological replicates with two technical replicates each (error bars = ± SD). Statistical significance is indicated (*=$p < 0.05$; ****=$p < 0.0001$).

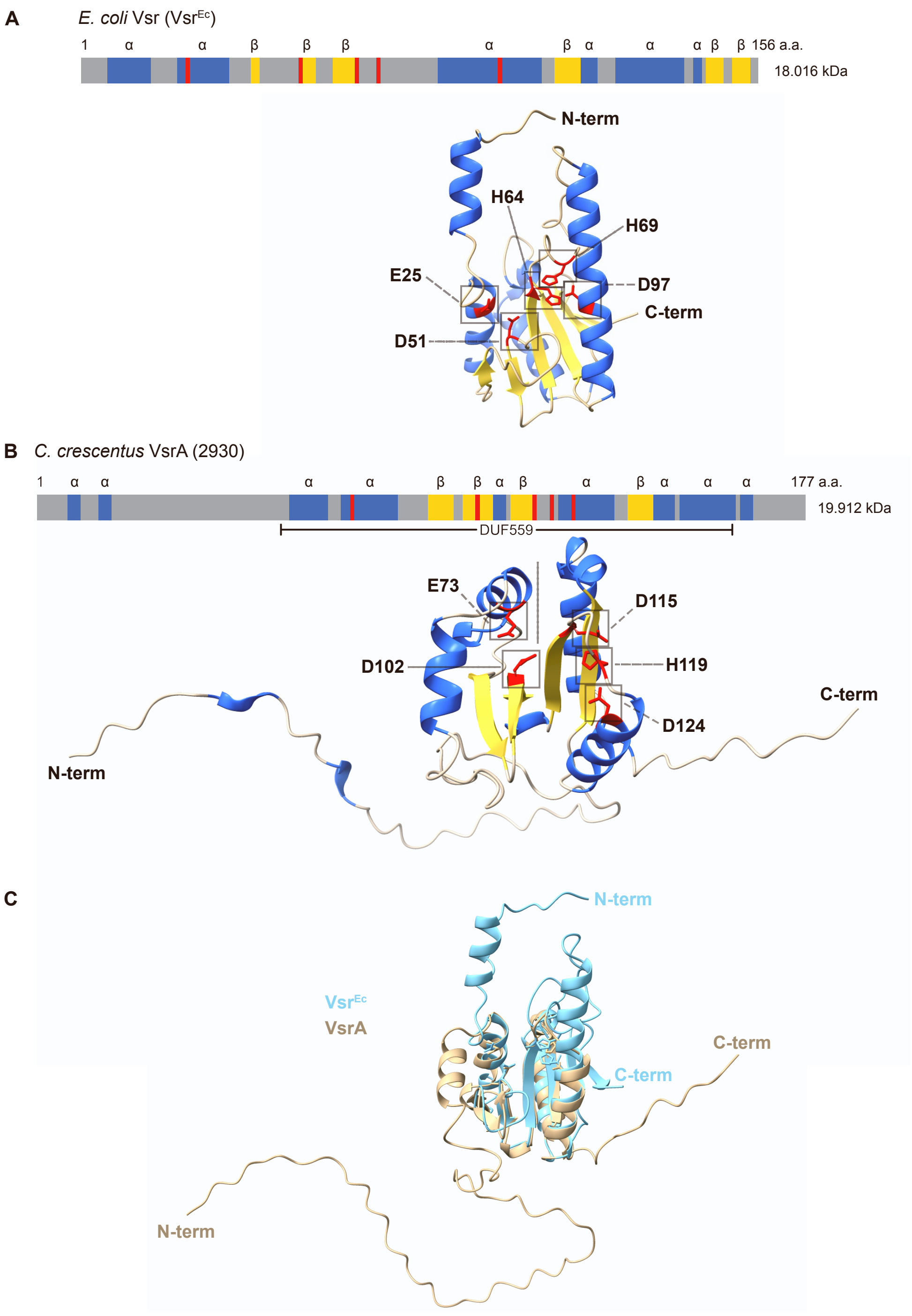

Figure S9: Comparison of the predicted structures of the *Escherichia coli* Vsr^{Ec} and the *Caulobacter crescentus* VsrA proteins. Predicted organizations, structures, length in amino-acids and molecular weights of the Vsr^{Ec} protein of the *E. coli* K12 strain **(A)** and of the VsrA (CCNA_02930) protein of the *C. crescentus* NA1000 strain **(B)** are displayed. In panels **A** and **B**, linear and 3D schematics of the predicted structures [2,3] are shown. β-strands and α-helices are shown in yellow and blue, respectively. The amino-acid residues that are important for the endonuclease activity of Vsr^{Ec} (E25, D51, H64, H69 and D97) [4,5] are highlighted in red. The equivalent residues identified on VsrA (E73, D102, D115, H119 and D124) are also highlighted in red. (**C**) Alignment of the two predicted 3d structures from (A) and (B) with VsrA in brown and Vsr^{Ec} in blue. This superimposition was done using ChimeraX v.1.8 [6].

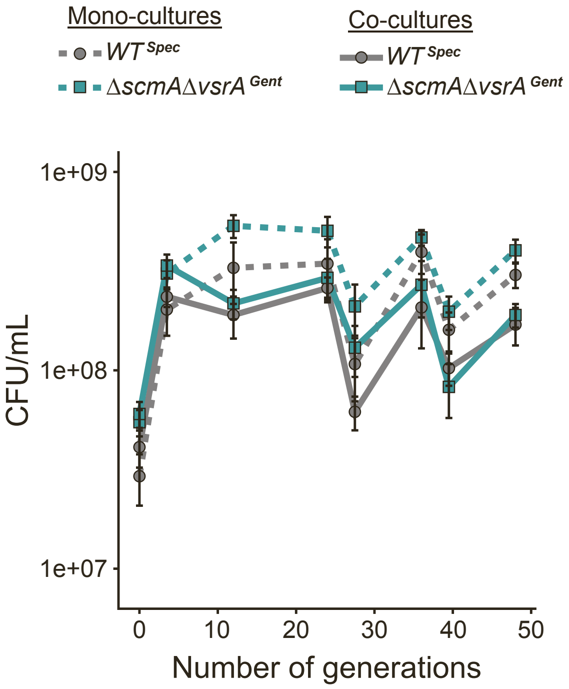

Figure S10: Wild-type *C. crescentus* cells do not outcompete *ΔscmA ΔvsrA* cells during competition experiments. Monocultures (dashed lines) and co-cultures (solid lines) of WT^{Spec} (JC2985) and/or Δ*scmA ΔvsrA*^{Gent} (JC3049) cells were cultivated in complex PYE medium for ~48 generations (the generation time of these strains was close to 2 hours under these conditions). Before each regular dilution, spectinomycin-resistant (WT^{Spec}) and gentamycin-resistant (Δ*scmA ΔvsrA*^{Gent}) colony forming units (CFU) per mL of (co-)cultures were measured on antibiotic-containing PYEA plates. The plotted values correspond to average measurements from four independent biological replicates (error bars = ± SD).

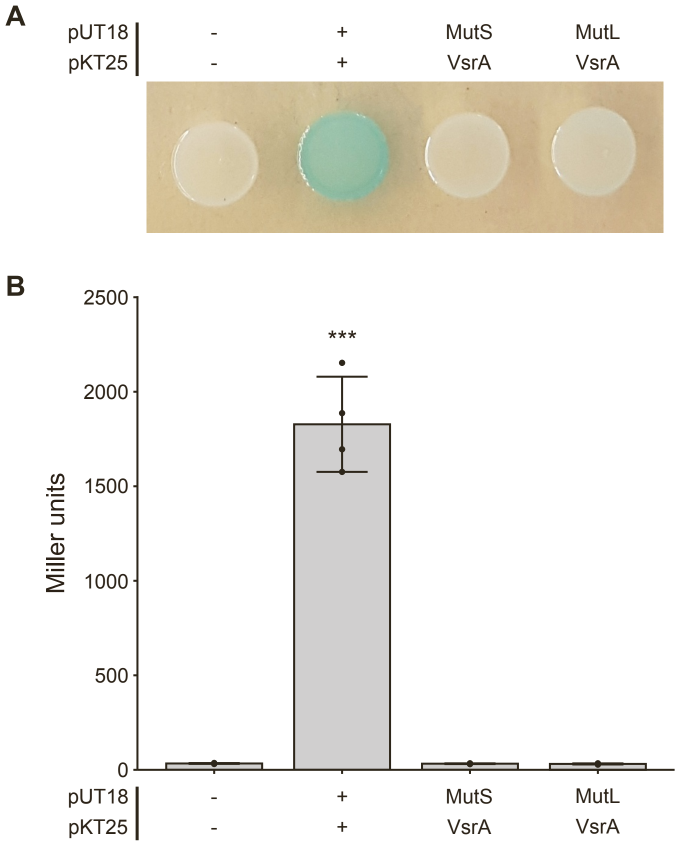

Figure S11: Bacterial two hybrid assays indicate that VsrA does not interact with the *C. crescentus* MutS or MutL proteins. MutS and MutL were fused to the T18 fragment of an adenylate cyclase, while VsrA was fused to a T25 fragment to test interactions. Assays were performed using *E. coli* BTH101 cells carrying either the empty pUT18 and pKT25 plasmids, or the pUT18-zip and pKT25-zip plasmids as negative (-) and positive (+) controls, respectively. (**A**) Representative images of bacterial patches on LBA+X-Gal+IPTG plates. (**B**) Results of β-galactosidase assays for BACTH using cells collected from 4 plates as shown in (A). The values are averages of activities measured from four independent biological replicates (error bars = ± SD). Statistical significance is indicated (***=$p < 0.001$).

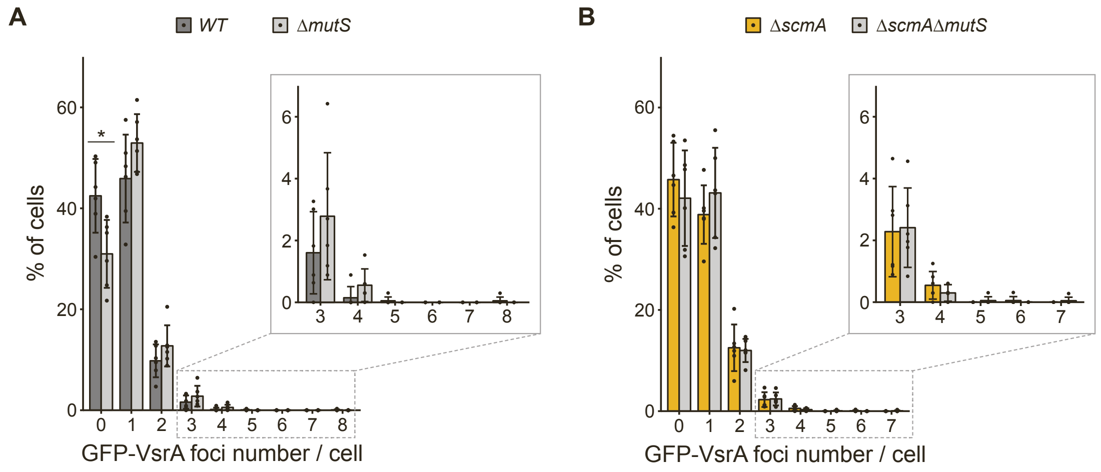

Figure S12: MutS has no impact on the proportion of cells displaying GFP-VsrA foci in stationary phase. The native *vsrA* gene was replaced by a *gfp-vsrA* construct in *WT*, Δ*mutS*, Δ*scmA* and Δ*scmA* Δ*mutS* cells, giving the JC2860, JC3072, JC2861 and JC3075 strains, respectively. These strains were then cultivated in PYE medium until they reached stationary phase before being imaged with a fluorescence microscope. Phase contrast and GFP images of cells were acquired and the number of detectable GFP-VsrA foci per cell was analyzed for each cell population. Minimum 300 cells were analyzed for each biological replicate. Panels **(A)** and **(B)** show the percentage of cells displaying a given number of GFP-VsrA foci per cell for each population. The plotted values are averages of at least three independent biological replicates. (error bars = ± SD). Statistically significant differences comparing cells with different genotypes using a student's *t*-test is indicated as follows: * = *P*-value < 0.05.

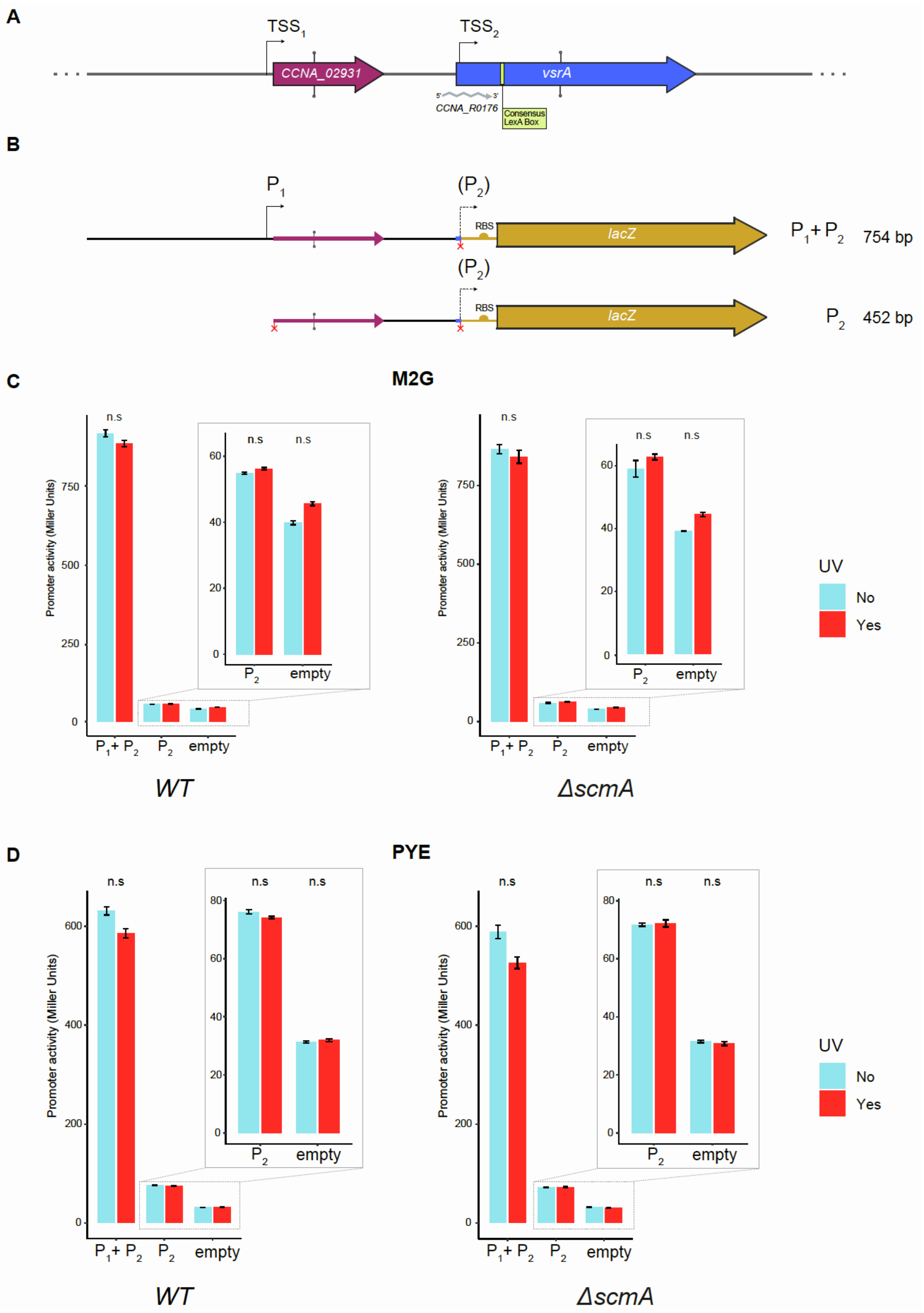
A
TSS1
TSS2
CCNA_02931
vsrA
CCNA_R0176
Consensus LexA Box
B
P1
(P2)
RBS
lacZ
P1 + P2
754 bp
P2
452 bp
C
M2G
n.s
Promoter activity (Miller Units)
P1 + P2
P2
empty
WT
ΔscmA
UV
No
Yes
D
PYE

Figure S13: *vsrA* is mostly transcribed from an active promoter upstream of *CCNA_02931*, which is neither activated during DNA damaging conditions, nor in *ΔscmA* cells. (A) Schematic showing the *vsrA* ORF (blue arrow) and its upstream region with the *CCNA_02931* ORF (burgundy arrow) on the *C. crescentus* genome. Potential TSS and the position of a putative "sense" ncRNA (named CCNA_R0176), both mapped from RNA-Seq data [7], are indicated. Lollipops indicate the position of YGCCGGCR motifs methylated by ScmA. The LexA box detected in this region (perfect match with the known GTTC(N7)GTTC LexA consensus from [8]) is also shown (yellow box). **(B)** Schematics showing the two chromosomal regions cloned upstream of the *lacZ* gene in the p*lacZ*290 vector (Promoter::*lacZ* transcriptional fusions). Red crosses indicate stop codons introduced in frame with the *vsrA* or *CCNA_02931* ORFs to prevent their translation. **(C)** β-galactosidase assays performed with cells cultivated in M2G minimal medium. **(D)** β-galactosidase assays performed with cells cultivated in PYE complex medium. For (C) and (D): The p*lacZ*290 derivatives (pP1+P2::*lacZ290* and pP2::*lacZ290)* were introduced into *WT* (JC450) and Δ*scmA* (JC2005) *C. crescentus* cells. The resulting strains were cultivated in the indicated media until cultures reached an $0.1<OD_{660nm}<0.2$ and half of each culture was then UV-irradiated (100J/m2 at 254nm as in [9] to induce an SOS response but using a Stratalinker UV Crosslinker Model 2400). Cells were then cultivated for 1.5-2 hours more (reaching $0.3<OD_{660nm}<0.4$) before β-galactosidase assays were performed. The plotted promoter activity values (in Miller units) are averages of 3 independent biological replicates. Error bars correspond to standard deviations (± SD). Statistical significance comparing cells exposed or not to UVs were evaluated using a student's *t*-test: ns = *P*-value > 0.05.

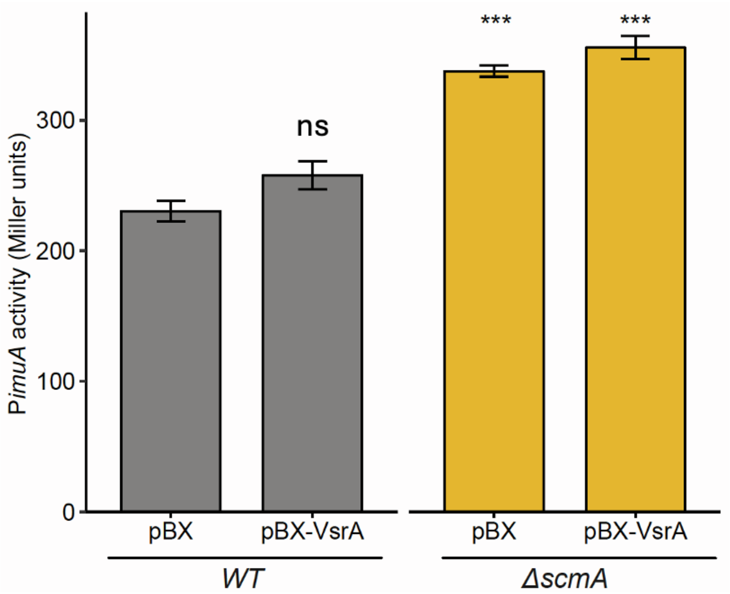

Figure S14: VsrA overproduction does not induce an SOS response. The pP*imuA*::*lacZ*290 plasmid (SOS reporter) and the pBX-VsrA plasmid (or the pBX empty control vector) were introduced into *WT* (JC450) and Δ*scmA* (JC2005) *C. crescentus* cells. The resulting strains were cultivated in M2G medium with 0.3% xylose until cultures reached an $0.15 < OD_{660nm} < 0.35$ and β-galactosidase assays were then performed. The plotted P*imuA* activity values (in Miller units) are averages of 2-3 independent biological replicates, each with two technical replicates. Error bars correspond to standard deviations (± SD). Statistically significant differences compared to *WT* cells with the pBX empty vector using a student's *t*-test is indicated as follows: ns = *P*-value > 0.05; *** = *P*-value < 0.001).

Supplementary Tables:

Table S1: Bacterial strains used in this study

Genotype	Description	Given name	Reference/Origin
Caulobacter crescentus			
NA1000 (WT)	Synchronizable derivative of wild-type strain CB15 (named NA1000 or CB15N)	JC450	[10]
NA1000 Δ*scmA*	NA1000 with *scmA* (*CCNA_01085*) deletion	JC2005	This study
NA1000 Δ*vsrA*	NA1000 deleted for *vsrA* (*CCNA_02930*)	JC2540	This study
NA1000 Δ*scmA* Δ*vsrA*	NA1000 Δ*scmA* deleted for *vsrA*	JC2542	This study
NA1000 Δ*mutS*	NA1000 deleted for *mutS* (*CCNA_00012*)	JC1427	[1]
NA1000 Δ*scmA* Δ*mutS*	NA1000 Δ*scmA* deleted for *mutS*	JC2987	This study
NA1000 Δ*mutL*	NA1000 deleted for *mutL* (*CCNA_00731*)	JC1426	[1]
NA1000 Δ*scmA* Δ*mutL*	NA1000 Δ*scmA* deleted for *mutL*	JC2474	This study
NA1000 Δ*2876*	NA1000 deleted for *CCNA_02876*	JC2539	This study
NA1000 Δ*scmA* Δ*2876*	NA1000 Δ*scmA* deleted for *CCNA_02876*	JC2541	This study
NA1000 P*imuA*::*mCherry*	NA1000 carrying a P*imuA::mCherry* transcriptional fusion at *imuA* native locus	JC3104	This study
NA1000 Δ*scmA* P*imuA*::*mCherry*	NA1000 Δ*scmA* carrying a P*imuA::mCherry* transcriptional fusion at *imuA* native locus	JC3105	This study
NA1000 Δ*vsrA* P*imuA*::*mCherry*	NA1000 Δ*vsrA* carrying a P*imuA::mCherry* transcriptional fusion	JC3107	This study
NA1000 Δ*scmA* Δ*vsrA* P*imuA*::*mCherry*	NA1000 Δ*scmA* Δ*vsrA* carrying a P*imuA::mCherry* transcriptional fusion at *imuA* native locus	JC3109	This study
NA1000 P*bapE*::*mCherry*	NA1000 carrying a P*bapE::mCherry* transcriptional fusion at *bapE* native locus	JC2813	This study
NA1000 Δ*scmA* P*bapE*::*mCherry*	NA1000 Δ*scmA* carrying a P*bapE::mCherry* transcriptional fusion at *bapE* native locus	JC2814	This study
NA1000 Δ*scmA* Δ*vsrA* P*bapE*::*mCherry*	NA1000 Δ*scmA* Δ*vsrA* carrying a P*bapE::mCherry* transcriptional fusion at *bapE* native locus	JC2815	This study
NA1000 *GFP::vsrA*	NA1000 carrying a *GFP::vsrA* translational fusion at *vsrA* native locus	JC2860	This study
NA1000 Δ*scmA* *GFP::vsrA*	NA1000 Δ*scmA* carrying a *GFP::vsrA* translational fusion at *vsrA* native locus	JC2861	This study
NA1000 Δ*mutS* *GFP::vsrA*	NA1000 Δ*mutS* carrying a *GFP::vsrA* translational fusion at *vsrA* native locus	JC3072	This study
NA1000 Δ*scmA* Δ*mutS* *GFP::vsrA*	NA1000 Δ*scmA* Δ*mutS* carrying a *GFP::vsrA* translational fusion at vsrA native locus	JC3075	This study

NA1000 SpecR	NA1000 with pVGFPC-1 inserted at the *vanA* locus to confer SpecR	JC2985	This study
NA1000 GentR	NA1000 with pVGFPC-4 inserted at the *vanA* locus to confer GentR	JC2983	This study
NA1000 Δ*scmA* GentR	NA1000 Δ*scmA* with pVGFPC-4 inserted at the *vanA* locus to confer GentR	JC2984	This study
NA1000 Δ*scmA* Δ*vsrA* GentR	NA1000 Δ*scmA* Δ*vsrA* with pVGFPC-4 inserted at the *vanA* locus to confer GentR	JC3049	This study
Escherichia coli			
F- *mcrA Δ(mrr-hsdRMS-mcrBC) φ80lacZΔM15 ΔlacX74 nupG recA1 araΔ139 Δ(ara-leu)7697 galE15 galK16 rpsL(Str^R) endA1λ$^-$*	Used for cloning procedures	TOP10	Invitrogen
F *cya*-99 *araD*139 *galE*15 *galK*16 *rpsL*1 (*Str^R*) *hsdR*2 *mcrA*1 *mcrB*1	Adenylate cyclase-deficient *E. coli* strain used for bacterial adenylate cyclase two-hybrid assays (BACTH)	BTH101	Euromedex

Table S2: Plasmids constructed and/or used during this study

**Plasmid name**	**Description**	**Strain name with this plasmid**	**Reference**
pBXMCS-2	Medium copy number vector with pBBR1 origin (Kan^R)	LS4421	[11]
pBX-1motif	pBXMCS-2 plasmid containing a 2200 bp insert flanked by HpaII sites and containing a single YGCCGGCR motif (CGCCGGCG) (KanR)	JC3051	This study
pBX-VsrA	pBXMCS-2 plasmid containing the *vsrA/CCNA_02930* ORF under the control of the P*xylX* promoter (KanR)	JC2673	This study
pNPTS138	Suicide vector carrying the *sacB* gene, with ColEI origin and *oriT* (KanR) used as a tool to delete/mutate genes in *C. crescentus* (cannot replicate in *C. crescentus*)	JC473	D. Alley, unpublished
pNPTS138::Δ*scmA*	pNPTS138 containing *scmA* flanking regions and used to create the *scmA* deletion (KanR)	JC1999	This study
pNPTS138::Δ*vsrA*	pNPTS138 containing *vsrA* flanking regions and used to create the *vsrA* deletion (KanR)	JC2496	This study
pNPTS138::Δ*mutS*	pNPTS138 containing *mutS* flanking regions and used to create the *mutS* deletion (KanR)	JC1282	[1]
pNPTS138::Δ*mutL*	pNPTS138 containing *mutL* flanking regions and used to create the *mutL* deletion (KanR)	JC1283	[1]

pNPTS138::Δ*2876*	pNPTS138 containing *CCNA_02876* flanking regions and used to create the *CCNA_02876* deletion (KanR)	JC2495	This study
pCHYC-1	Vector with ColEI origin (not functional in *C. crescentus*) and *oriT* (SpecR, StrepR) used to generate C-terminal mCherry protein fusions and used as template to amplify *mCherry* to construct pNPTS138::P*imuA::mCherry*	LS4218	[11]
pNTPS138::P*imuA::* *mCherry*	pNPTS138 derivative used to insert *mCherry* under the control of the native *imuA* promoter in the *C. crescentus* chromosome (KanR)	JC3099	This study
pNTPS138::P*bapE::* *mCherry*	pNPTS138 derivative used to insert a*mCherry* under the control of the native *bapE* promoter in the *C. crescentus* chromosome (KanR)	JC2792	This study
pGFPC-1	Vector with ColEI origin (not functional in *C. crescentus*) and *oriT* used to amplify the *GFP* gene to construct pNPTS138::*GFP::vsrA* (SpecR, StrepR)	LS4213	[11]
pNPTS138::*GFP::vsrA*	pNPTS138 derivative used to insert *GFP::vsrA* at the native *vsrA* locus (KanR)	JC2852	This study
pVGFPC-1	Vector with ColEI origin (not functional in *C. crescentus*) and *oriT* and with the *vanA* promoter used to confer SpecR resistance once integrated at the native *vanA* locus on the *C. crescentus* chromosome (SpecR, StrepR)	LS4229	[11]
pVGFPC-4	Vector with ColEI origin (not functional in *C. crescentus*) and *oriT* and with the *vanA* promoter used to confer GentR resistance once integrated at the native *vanA* locus on the *C. crescentus* chromosome (GentR)	LS4340	[11]
p*lacZ*290	Low copy number vector with RK2 origin and *oriT,* used to create *lacZ* transcriptional fusions (Tet^R)	JC452	[12]
pP*imuA*::*lacZ*290	p*lacZ*290 derivative with *imuA* (*CCNA_03319*) promoter inserted upstream of *lacZ* (TetR)	JC1053	[13]
pP1+P2::*lacZ290*	p*lacZ*290 derivative with the 754 bp promoter region upstream of the *vsrA/CCNA_02930* ORF inserted upstream of *lacZ* (TetR)	JC3419	This study
pP2::*lacZ290*	p*lacZ*290 derivative with the 452 bp promoter region upstream of the *vsrA/CCNA_02930* ORF inserted upstream of *lacZ* (TetR)	JC3418	This study
pUT18	High copy number vector with ColEI origin, used for BACTH C-terminal T18 fusions (AmpR)	JC2135	Euromedex
pKT25	Medium copy number vector with p15A origin, used for BACTH C-terminal T25 fusions (KanR)	JC2138	Euromedex

pUT18.zip	pUT18 encoding T18-leucine zipper from yeast GCN4 (AmpR)	JC2137	Euromedex
pKT25-zip	pKT25 encoding T25-leucine zipper from yeast GCN4 (KanR)	JC2139	Euromedex
pUT18::*mutS*	pUT18 encoding MutS-T18 (AmpR)	JC3030	This study
pUT18::*mutL*	pUT18 encoding MutL-T18 (AmpR)	JC3031	This study
pKT25::*vsrA*	pKT25 encoding VsrA-T25 (KanR)	JC3038	This study

Table S3: Oligonucleotides used during this study. Relevant restriction sites of indicated restriction endonucleases are underlined.

Primer name	Sequence (5' to 3')	Used for
NM210	AATAAGCTTCCGGCCACAAGCCGATCGTC (HindIII)	construction of pBX-1motif
NM211	AAAAGGTACCAGAACGACGCCGGCG (KpnI)	
NM212	ATGGTACCCTCAGGTCGATCAGGCTG (KpnI)	
NM213	ATACTAGTCCGGGAATCCGCAGC (SpeI)	
NM91	ATACATATGTGTCCGGGCGATGG (NdeI)	Construction of pBX-VsrA
NM92	ATAGAATTCTCATCCGTCGACTGCGTCG (EcoRI)	
TC79	TCACTAGTATGTCGATCCTGGTCAACAGCGA (SpeI)	construction of pNPTS138::Δ*scmA*
TC80	GCTGGATCCCACCAATTCCACTCCAAAGTCGA (BamHI)	
TC81	CGAGGATCCGCCTAGATTTCCATGGGTCGGAT (BamHI)	
TC82	CTCGAATTCCCCGCGCTTAAACTGCAATCCG (EcoRI)	
NM31	GGACTAGTGCAGAGAGGACATTGTCCATGC (SpeI)	construction of pNPTS138::Δ*vsrA*
NM32	GGAGCGGCGCAGGCTTCAAGTTAGCAAGCTGCAAAGTG	
NM33	GCCCCCCACTTTGCAGCTTGCTAACTTGAAGCCTGCGCC	
NM34	ATGGATCCTGACCTTCCGGGTGGGAACCTG (BamHI)	
NM27	GGACTAGTTTGCCGAAAAACGCCTCCATC (SpeI)	construction of pNPTS138::Δ*2876*
NM28	CAAATTTTCCGCAGTTAGGGTTATTCGGGTGGCGACATC	
NM29	CGAGAGATGTCGCCACCCGAATAACCCTAACTGCGGAAA	
NM30	CGGGATCCGTTGAAGAGGCCGACTTATTTC (BamHI)	
NM218	GGACTAGTTGGGCTGACAGATCGGAAG (SpeI)	construction of pNPTS138::P*imuA::mCherry*
NM219	TCTCCTCTTTAATTTATCCGAAGCGTCGTCCGG	
NM220	ACGCTTCGGATAAATTAAAGAGGAGAAATACTAGATGGTGAGCAAGGGCGAGGA	
NM221	CTTGGGGGGAGGATTTACTTGTACAGCTCGTCCATG	
NM222	GAGCTGTACAAGTAAATCCTCCCCCCAAGGGGGAGGAG	
NM223	ATAGAATTCGGCGCAATGGCGAGGTCC (EcoRI)	
NM114	GGACTAGTGTTAGCCATCGCGCTTTTCTG (SpeI)	
NM115	TCTCCTCTTTAATTCATCGCGACCAGCAGGCGA	

NM116	CTGGTCGCGATGAATTAAAGAGGAGAAATACTAGATGGTGAGCAAGGGCGAGGA	construction of pNPTS138::P*bapE::mCherry*
NM117	GTGCCTAAGCCCCTTTACTTGTACAGCTCGTCCATG	
NM118	GAGCTGTACAAGTAAAGGGGCTTAGGCACCCTTGG	
NM119	ATA<u>GAATTC</u>GGATATCCTTCACCACCCGCTCAC (EcoRI)	
NM169	GG<u>ACTAGT</u>CTGGAGGTTAACGCGTGGCA (SpeI)	construction of pNPTS138::*GFP::vsrA*
NM170	CTTGCTCACCATGCCCGGAAGGGATGGATAGG	
NM171	TATCCATCCCTTCCGGGCATGGTGAGCAAGGGCGAGGA	
NM172	CGGACAACCTCCACCAGCTGCAGCCTTGTACAGCTCGTCCATG	
NM173	TGCAGCTGGTGGAGGTTGTCCGGGCGATGGGCTC	
NM174	ATA<u>GAATTC</u>GCGCAGGCTTCAAGGATCAG (EcoRI)	
NM201	AAAAAA<u>GGATCC</u>CAACGCCCACGCCACGCC (BamHI)	construction of pUT18::*mutS*
NM202	AA<u>GGTACC</u>CGGGCCGTGAGCAGACCCTTAAG (KpnI)	
NM203	AAAA<u>GGATCC</u>CCCCATCCGCCGCCTGCC (BamHI)	construction of pUT18::*mutL*
NM204	AAAA<u>GGTACC</u>CGCCGCCGCCCGAACAGCTTCTC (KpnI)	
NM205	AAAA<u>GGATCC</u>CTGTCCGGGCGATGGGCTC (BamHI)	construction of pKT25::*vsrA*
NM206	AAAA<u>GGTACC</u>CGTCCGTCGACTGCGTCGACAC (KpnI)	
NPJC30	ACTG<u>AAGCTT</u>TTACGGGCGGAGCCCATC (HindIII)	construction of pP1+P2::*lacZ290* and pP2::*lacZ290*
NPJC31	CTCA<u>GAATTC</u>ACATAGTCCATGCAGGCCCT (EcoRI)	construction of pP2::*lacZ290*
NPJC32	CTCA<u>GAATTC</u>GCGAACCATGTCGAGCCTGT (EcoRI)	construction of pP1+P2::*lacZ290*

Supplementary References:

1. Chai, T., Terrettaz, C., and Collier, J. (2021). Spatial coupling between DNA replication and mismatch repair in *Caulobacter crescentus*. Nucleic Acids Res *49*, 3308-3321. 10.1093/nar/gkab112.
2. Jumper, J., Evans, R., Pritzel, A., Green, T., Figurnov, M., Ronneberger, O., Tunyasuvunakool, K., Bates, R., Zidek, A., Potapenko, A., et al. (2021). Highly accurate protein structure prediction with AlphaFold. Nature *596*, 583-589. 10.1038/s41586-021-03819-2.
3. Varadi, M., and Velankar, S. (2023). The impact of AlphaFold Protein Structure Database on the fields of life sciences. Proteomics *23*, e2200128. 10.1002/pmic.202200128.
4. Tsutakawa, S.E., Jingami, H., and Morikawa, K. (1999). Recognition of a TG mismatch: the crystal structure of very short patch repair endonuclease in complex with a DNA duplex. Cell *99*, 615-623. 10.1016/s0092-8674(00)81550-0.
5. Tsutakawa, S.E., Muto, T., Kawate, T., Jingami, H., Kunishima, N., Ariyoshi, M., Kohda, D., Nakagawa, M., and Morikawa, K. (1999). Crystallographic and functional studies of very short patch repair endonuclease. Mol Cell *3*, 621-628. 10.1016/s1097-2765(00)80355-x.
6. Meng, E.C., Goddard, T.D., Pettersen, E.F., Couch, G.S., Pearson, Z.J., Morris, J.H., and Ferrin, T.E. (2023). UCSF ChimeraX: Tools for structure building and analysis. Protein Sci *32*, e4792. 10.1002/pro.4792.
7. Bharmal, M.H., Aretakis, J.R., and Schrader, J.M. (2020). An Improved *Caulobacter crescentus* Operon Annotation Based on Transcriptome Data. Microbiol Resour Announc *9*. 10.1128/MRA.01025-20.
8. da Rocha, R.P., Paquola, A.C., Marques Mdo, V., Menck, C.F., and Galhardo, R.S. (2008). Characterization of the SOS regulon of *Caulobacter crescentus*. J Bacteriol *190*, 1209-1218. JB.01419-07 [pii]
10.1128/JB.01419-07.
9. Modell, J.W., Hopkins, A.C., and Laub, M.T. (2011). A DNA damage checkpoint in *Caulobacter crescentus* inhibits cell division through a direct interaction with FtsW. Genes Dev *25*, 1328-1343. 10.1101/gad.2038911.
10. Evinger, M., and Agabian, N. (1977). Envelope-associated nucleoid from *Caulobacter crescentus* stalked and swarmer cells. J Bacteriol *132*, 294-301.
11. Thanbichler, M., Iniesta, A.A., and Shapiro, L. (2007). A comprehensive set of plasmids for vanillate- and xylose-inducible gene expression in *Caulobacter crescentus*. Nucleic Acids Res *35*, e137. gkm818 [pii]
10.1093/nar/gkm818.
12. Gober, J.W., and Shapiro, L. (1992). A developmentally regulated *Caulobacter* flagellar promoter is activated by 3' enhancer and IHF binding elements. Mol Biol Cell *3*, 913-926.
13. Galhardo, R.S., Rocha, R.P., Marques, M.V., and Menck, C.F. (2005). An SOS-regulated operon involved in damage-inducible mutagenesis in *Caulobacter crescentus.* Nucleic Acids Res *33*, 2603-2614. 33/8/2603 [pii]
10.1093/nar/gki551.
