# Supplementary material for: Cytosine methylation contributes to the fitness of Caulobacter cells naturally expressing a Vsr-like protein
Source: iScience. 2026 Jan 20;29(2):114749. doi: 10.1016/j.isci.2026.114749 (PMC12907096; doi:10.1016/j.isci.2026.114749)
Supplement: Data S1. Interactive volcano plot corresponding to RNA-seq results comparing the transcriptome of ΔscmA ΔvsrA (JC2542) and ΔvsrA (JC2540) cells, related to Figure 3B — Black and red dots correspond to genes that were significantly misregulated (adjusted p value < 0.05 and log2FC ≥ 1 in red for “upReg” or log2FC ≤ −1 in black for “downReg”); gray dots correspond to genes that were not significantly misregulated (“nonDE”: adjusted p value > 0.05 or −1<log2FC < 1). [file mmc4.zip › renamed_bfe88.html]

glimmaXY
